# Challenges to mangroves of the Semiarid Equatorial Coast of Brazil in the Anthropocene

**DOI:** 10.1017/cft.2024.16

**Published:** 2024-12-04

**Authors:** Luiz Drude de Lacerda, Alexander Cesar Ferreira, Rebecca Borges, Raymond Ward

**Affiliations:** 1 Instituto de Ciências do Mar, Universidade Federal do Ceará, Fortaleza, Ceará, Brazil; 2 Alfred Wegener Institute Helmholtz Centre for Polar and Marine Research, Bremerhaven, Germany; 3 Helmholtz Institute for Functional Marine Biodiversity, University of Oldenburg (HIFMB), Oldenburg, Germany; 4 School of Geography, Queen Mary, University of London, London, UK

**Keywords:** forest structure, biomass, soil carbon, functional groups, anthropogenic drivers, climate change

## Abstract

The semiarid northeast coast of Brazil harbours just less than 44,300 ha of mangroves, 4% of Brazilian total. Notwithstanding this relatively small area, these forests have high ecological and economic importance, sustaining traditional fisheries and protecting biodiversity, including many threatened species. They present unique biogeochemical characteristics resulting in distinct ecosystem functioning compared to mangroves located in humid areas. Semiarid mangroves present lower aboveground biomass compared to humid region mangroves but show similar belowground biomass. Whereas mangrove soils in humid areas are strongly influenced by sulphate reduction, iron geochemistry is a primary driver of soil characteristics in semiarid mangrove soils, suggesting different responses to climate change drivers between them. Although legally protected, they have incurred continuous degradation due to regional drivers, mostly aquaculture and river damming, which differs from those in humid coast mangroves. Semiarid mangroves are also particularly sensitive to drivers associated with global climate change (high temperatures, reduced rainfall and sea level rise). These conditions occur at a global scale; however, the impacts are worsened by the natural conditions of semiarid coastlines, which already provide biologically stressful conditions for mangroves. This article compares the impacts of such drivers in semiarid mangroves with those of humid mangroves, focusing on their biogeochemical response and eventual rehabilitation.

## Graphical abstract

**Figure:**
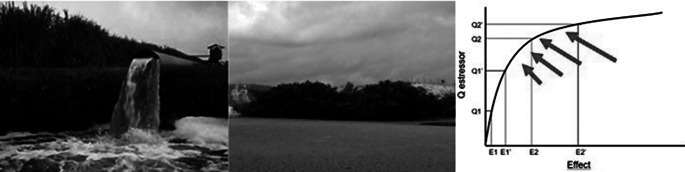

## Impact statement

Semiarid mangroves in Brazil cover a small extent compared to humid areas, but with high ecological and economic importance that sustain traditional fisheries and high biodiversity, including many threatened species. Although with lower aboveground biomass, they show similar below ground biomass and soil carbon stocks compared to humid mangroves. Their structure and functioning results from interactions among functional groups of organisms, that strongly influences key ecological processes, but are presently affected by anthropic and climatic factors. Soil biogeochemical mediator microbiota, burrowers/bioturbators and herbivores/detritivores, are the main affected groups, leading to functional degradation and eventual dieback. Notwithstanding legal protection in Brazil, semiarid mangroves witness progressive degradation due to regional drivers, mostly aquaculture and river damming, a worldwide scenario in semiarid coasts. Semiarid mangroves are particularly sensitive to drivers associated with global climate change that are worsened by the natural biologically stressful conditions. Landward migration as a response to climate change is constrained by dunes encroachment and urban expansion. The main strategy to conserve ecosystem services from semiarid mangroves is to preserve the forests. Assisted recovery of degraded sites was successful in most cases and bring back rapidly the crucial ecosystem functions, mostly when used native species with higher recovery capacity after impacts from natural or human-originated events. In Brazil, whilst most of these mangroves are protected, the levels of protection can, in practice, be quite weak, and management should be conducted in partnership with local communities, who rely heavily on mangroves goods and services, as well as spiritual and cultural history, often unaccounted for in policy and management.

## Mangroves of the Brazilian Equatorial Margin: extent and forest structure

**Figure 1.**
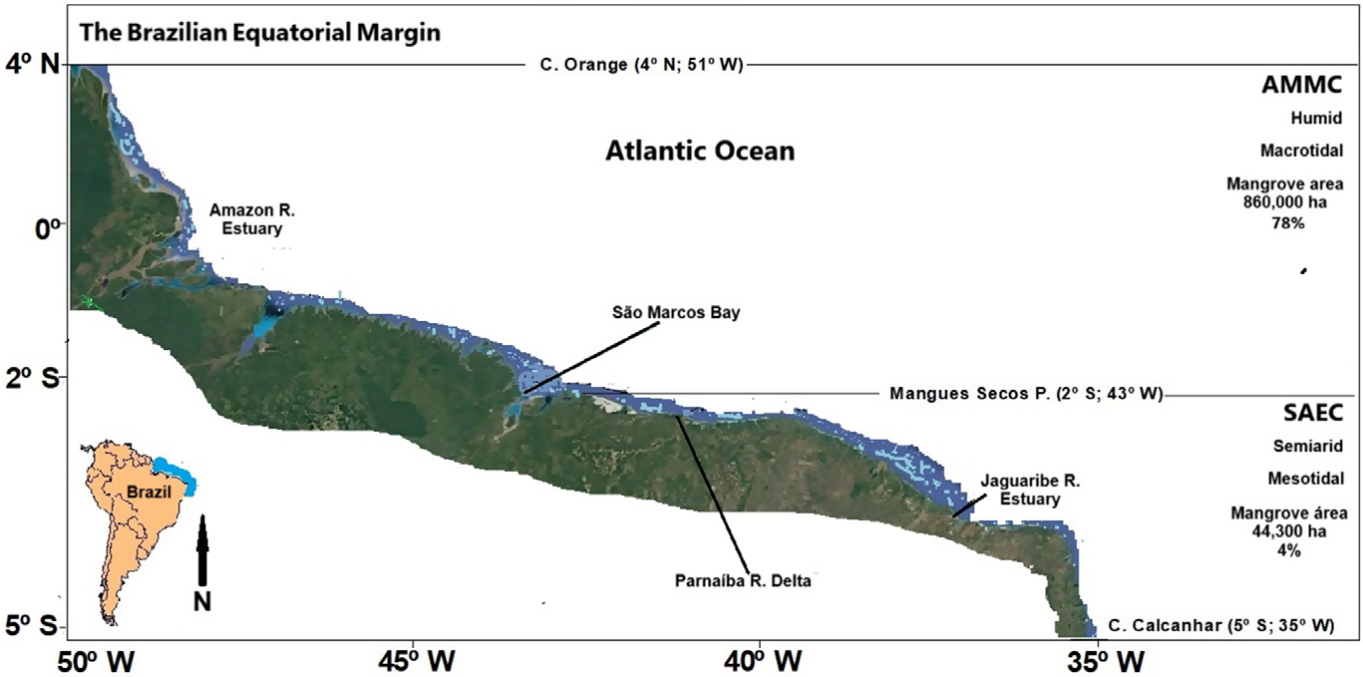
Location of mangrove forests along the humid and semiarid sectors of the Equatorial Brazilian Coast and their approximate forest area and the relative contribution (%) to the total mangrove area in Brazil. AMMC = Amazon Macrotidal Mangrove Coast; SAEC = Semiarid Equatorial Coast.

Brazil has the largest mangrove forest extent in the Americas and second largest worldwide. The most recent mangrove survey in Brazil suggests a total varying from 1,107,200 ha (Bunting et al. 2018) to 1,398,900 ha (ICMBio 2018), depending on the resolution of their mapping. Mangroves occur unevenly distributed along almost the entire Brazilian coast (Figure 1) and present distinct biological and ecological characteristics, depending on climate, fluvial contribution and the geomorphology of the littoral (Lacerda et al. 2022a). The Brazilian Equatorial Quaternary Margin borders two large marine ecosystems: the Semiarid Equatorial Coast (SAEC) to the east and the Amazon Macrotidal Mangrove Coast (AMMC) to the west. The two sectors have witnessed increasing environmental pressure from local anthropogenic and global climate change. Mangroves from the two sectors also share some characteristics as a narrow latitudinal distribution (<10°), mean annual temperature and the dominant tree species. However, they differ significantly in rainfall quantities and seasonal distribution, hydric stress and terrigenous supply of sediments. These differences are major controlling parameters of mangrove response to climate change and suggest SAEC mangroves as more vulnerable to environmental pressures. This work reviews the major mangrove processes affected by climate change and how they specifically affect SAEC mangroves and their ecosystems’ response. Eventually, due to the mentioned similarities, comparisons with the AMMC are used to better dimension the impacts on SAEC mangroves.

The SAEC extends by 1,038 km from the Mangues Secos Point in Maranhão State (2°S, 43°W) to Cape Calcanhar in Rio Grande do Norte State (5°S, 35°W), under tropical semiarid climate with mean annual temperature of ~26^o^C, annual rainfall varying from 400 to 900 mm and limited continental runoff (~590 m^3^ s^−1^) originated from highly seasonal and non-perennial rivers (Soares et al. 2021). Mangroves in the SAEC cover 44,300 ha, about 4% only of mangrove cover in Brazil, mostly as scattered forests or dwarf and scrubby stands (Diniz et al. 2019). The water source is sea water, variably diluted by rainwater and small rivers, presenting high seasonality and site-specificity and strongly dependent on human water use upstream of the hydrographic basins (Marins et al. 2002). Between 1980 and 2000, an increase in SAEC mangrove area of about 20% has been reported (Maia et al. 2006).

The AMMC extends from Cape Orange at 4^o^N 51^o^W to Mangues Secos Point in Maranhão State (2°S, 43°W). It comprises broad, lowland coastal plains and has a large fluvial contribution of about 135,000 m^3^ s^−1^ and inputting over 10^9^ tons year^−1^ of sediments. It has a tropical humid climate, high and constant annual average temperature (~27^o^C) and rainfall (~2,000 mm), a macrotidal regime (>7 m) and is bordered by a broad continental shelf extending 90–250 km. The enormous amount of sediment transported by Amazon basin rivers forms mud flats 20–30 km in width (Proisy et al. 2009), serving as sites for mangrove colonisation (Proisy et al. 2009). The AMMC harbours 78% of Brazil’s mangroves (~860,000 ha), including the largest (about 700,000 ha) continuous high-density mangrove forest in the world with little change in extent (<2%; 20,000 ha), over the past three decades (Kjerfve and Lacerda 1993; Diniz et al. 2019).

Salinity and freshwater supply differ by two to three orders of magnitude between the AMMC and SAEC subregions of the Equatorial Margin of Brazil and are crucial factors regulating mangrove growth, since temperature and solar radiation intensity are relatively similar. The strong seasonality of the SAEC induces high variations in soil and porewater salinity, which may reach values well above the local seawater salinity during the dry season (Marins et al. 2003). The infiltration of flood water from the rivers during the wet season keeps soil and porewater salinity lower than seawater. Trees and consequently forest structure reflect this stressor (Komiyama et al. 2019). At the AMMC, even low seasonal flow is sufficient to keep salinity lower than seawater. As a result, growth of mangrove trees is restricted during the dry season in SAEC, while no constraint to growth occurs in the humid subregion. Along the AMMC the large semidiurnal tidal amplitude, which may exceed 8 m in some places, allows the development of broad mangrove fringes of up to 40 km wide, and the large annual rainfall of more than 2,000 mm and abundant nutrient and freshwater enhances mangrove growth. *Avicennia* trees are particularly well-developed and can reach 40–45 m in height and up to 1.0 m in trunk diameter. SAEC, although with significant tidal amplitude (up to 4 m), lacks permanent freshwater and nutrient supply from the continent, restricting mangroves to a narrow strip along estuaries, migrating upstream along rivers depending on the extension of the saline intrusion. *Rhizophora mangle* is the most conspicuous species with heights that seldom exceed 10 m (Kjerfve and Lacerda 1993). Salt flats are a common feature in the SAEC mangroves due to strong evapotranspiration, and groundwater salinity can reach three times that of normal seawater, strongly affecting tree growth and producing stunted forests, mainly of *Avicennia* spp.

In the AMMC, freshwater macrophytes and flooded forest trees and palms invade the transition zone in the upper estuary, whereas in the SAEC, the presence of sand spits and relic dunes at the landward border of mangroves results in invasion by dry coastal ecosystem plants, mostly grasses, but also some associates, such as shrubs like *Conocarpus erectus* (buttonwood mangrove) and *Hibiscus* spp., that can only tolerate a small degree of flooding, but can occupy these seldom waterlogged high elevation areas. The landward edge of SAEC mangroves presents high diversity of herbs, sedges and grasses, mostly from the Poacea, Aizoaceae and Amaranthacea families (Silva et al. 2020).

**Figure 2.**
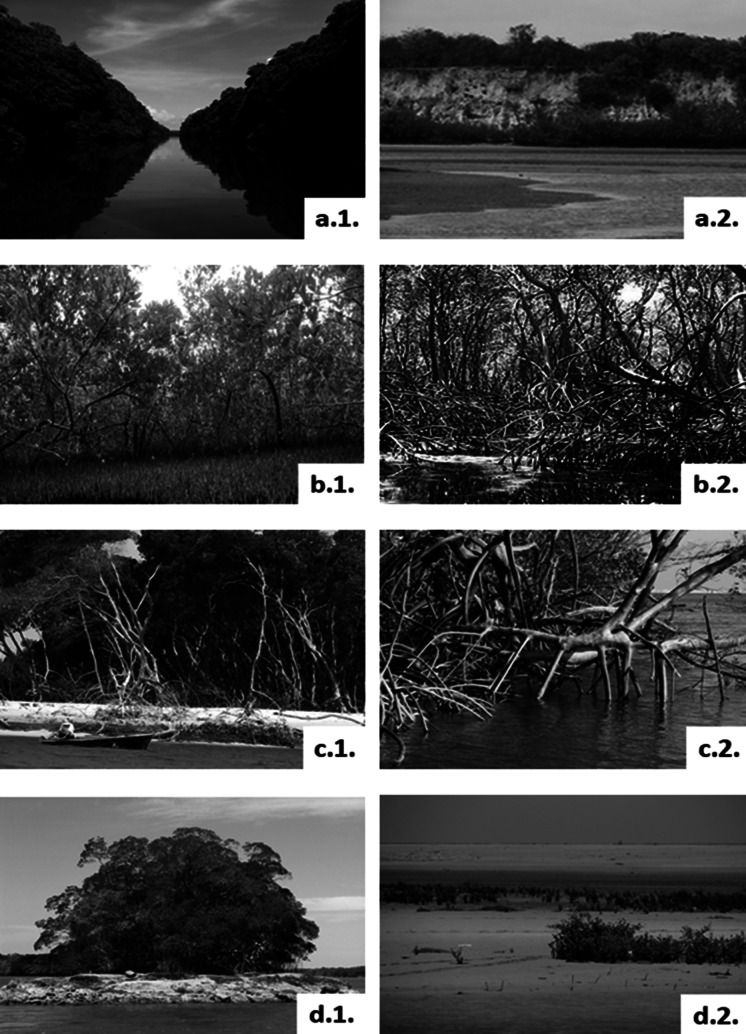
Examples of major mangrove forest types from the Semiarid Equatorial Coast (SAEC) of Brazil. a.1. Riverine forest bordering the Cocó River with the Metropolitan area of Fortaleza city; a.2. Narrow riverine forest limited by the Barreiras Formation in the Jaguaribe river estuary; b.1. Basin forest with high density of pneumatophores from *Avicennia* sp.; b.2. Basin forest with abundant Rhizophora mangle at the edge of a tidal creek and *Avicennia* sp. in the back; c.1.Partially eroded fringe forest at the Jaguaribe River mouth; c.2. aerial roots of *R. mangle* fringing the coast at the Jaguaribe river mouth; d.1. Overwash forest in an estuarine island at the Mundaú river; d.2. Overwash forest growing in newly formed sand banks at the Jaguaribe estuary.

Table 1, adapted from Lacerda et al. (2022a), summarises the major physical and structural characteristics of mangrove forests in the SAEC of Brazil, while Figure 2 shows examples of typical forest formations. In summary, four types of forests can be identified along the SAEC: Riverine; Basin; Fringe and Overwash. This division is mainly based on their specific location, but eventually results in different functional and structural attributes.

**Table 1.** A simplified characterization of mangrove forest types in the Semiarid Equatorial Coast (SAEC) of Brazil, modified from Lacerda et al. (2022a)

Type	Sediment origin	Geomorphology	Forest architecture
**Riverine**	Terrigenous from fluvial transport, clastic and siliciclastic sediments; highly seasonal; reworked eroded sediments	Exclusive to estuaries and deltas absent from open coasts; frequently eroded at the mouth of estuaries; river-front fringe trees frequently felled by winds	Short (<10 m height) trees, exceptionally up to 17 m, with low to intermediate aerial biomass; *Rhizophora mangle* as most frequent species; high tree density
**Basin**	Terrigenous from fluvial transport; clastic and marine organo-clastic particles from tidal flow	Lowlands behind riverbanks and fringing forests	Varying in height (3–10 m), exceptionally up to 18 m, *Avicennia* spp. as most frequent species; with intermediate to low biomass; tidal dominated; high soil carbon content; strongly reducing sediments
		Salt flats behind or within mangrove forests	Stunted ‘dwarf’ trees (<3 m); mostly of *Avicennia* spp.; high soil salinity
**Fringe**	Mostly remobilized, marine siliciclastic sediments, carbonates and bioclastic sands; strong influence of aeolian depositional processes	Bordering tidally dominated small river estuaries, frequently eroded	Varying in size; taller trees absent; intermediate to low biomass and soil carbon content; *Laguncularia racemosa* is a frequent species in fresh deposited sediments
		Lagoonal (with bioclastic sediments)	Short (<3 m height) trees; low biomass; and soil carbon content
**Overwash**	Marine carbonates and bioclastic sands; fluvial sands in river islands	Open waters sand banks and river islands	Short (<3 m height) trees; *R. mangle* is quite conspicuous at the border, while *Laguncularia racemosa* abounds in the interior

Riverine mangrove along the SAEC occurs along estuaries and are absent from open coasts, in contrast to the extensive and broad stretches of mangroves along the AMMC where they attain complex forest structure and high biomass. Along the SAEC, riverine stands occur in narrow fringes (Figure 2a.1, a.2) along rivers and estuaries margins, typically presenting lower canopy density and overall biomass that are frequently eroded, and trees are blown down by the wind. Well-developed fringe forests occur along the broad mud flat expanse of the Amazon coast, which provides protection from high-energy marine influences. In the SAEC, strong ocean forcing, waves and currents, as well as year-round stronger winds, readily remove any pioneer fringe forests along the open coast (Figure 2c.1), restricting them to the relatively protected waters of estuarine mouths (Figure 2c.2) and within the littoral fringes of coastal lagoons.

Basin forests (Figure 2b.1, b.2) typically occupy the landward portion of mangroves, where flooding occurs mostly during spring tides leading to high soil salinity due to strong evaporation. These extreme conditions result in high organic matter accumulation in soils, but from a poorly structured stunted vegetation, frequently dominated by the salt excreting *Avicennia* spp.

Overwash forests occur in recently formed fluvial and estuarine islands (Figure 2d.1, d.2) that generally present medium to coarse sands (Lacerda et al. 2007; Godoy and Lacerda 2014). There, they are flooded daily by tides with slow accumulation of organic matter in soils, most of the litterfall being exported to adjacent waters. This forest type sometimes also appears colonising beach rock outcrops at the intertidal level.

## Biology and ecology of functional groups (FGs)

Mangrove ecosystem properties, such as diversity, structural complexity, productivity and biomass, are influenced by local abiotic factors, for example, coastal geomorphology, rainfall, tidal amplitude, temperature, salinity and soil characteristics (nutrients and oxygen content, grain size, humidity) (Krauss et al. 2008), as well as biotic factors, like soil micro and macro-organism communities’ composition, bioturbation, propagules fixation ability, pollination and herbivory (Cannicci et al. 2021; Kristensen 2008; Ferreira et al. 2015). On the other hand, several attributes of mangrove community structure and function evolved from interactions among organisms, that is, among their niches.

Organisms of different species can perform similar ecological functions or ecosystem processes (independently of their taxonomic position) through ‘functional groups’ (FGs) (Blondel 2003). Thus, key FGs strongly influence mangrove structure and function, which can influence responses to anthropogenic drivers and environmental changes, but reciprocally, these changes can affect FGs ecological roles (Ferreira et al. 2024). Considering the structural resistance and resilience of the forests as crucial features to face climate change and human degradation drivers in semiarid mangroves, the most significant FGs are the biogeochemistry mediators (which include decomposers), bioturbators/burrowers, herbivores and wood borers (Ferreira et al. 2024).

### Biogeochemistry mediators

Microorganisms are an extremely important group of organisms in mangrove ecology and functionality (Holguín et al. 2001; El-Tarabily et al. 2021; Lacerda et al. 2022a; Farrer et al. 2022). A wide range of bacterial groups are involved in cycling of carbon (C), nutrients and several metals in mangrove soils, with some groups involved in more than one cycle, for example, the nitrogen (ammonification, nitrification, denitrification), sulphur (sulphate reduction), iron (Fe) and phosphorus (P) cycles (Bashan and Holguín 2002; Alongi 2021; Ferreira et al., 2022a). Bacteria and fungi can reach around 90% of the total microbial biomass, which also includes algae and protozoa and microphytobenthos, such as Diatomacea and Cyanobacteria, that contribute to significant amounts of buried carbon and trace elements in mangrove soils and in hypersaline tidal flats (Brown et al., 2021a; Lacerda et al. 2022b).

Mangroves are highly dependent on the efficiency of specific microbial communities both in soil and waters. The microbiome of SAEC mangroves is highly diverse and displays a higher level of complexity than those in the AMMC (see e.g. Andreote et al. 2012; Tavares et al. 2021). SAEC datasets extracted from *Rhizophora* roots environment were dominated by Proteobacteria (reducing nitrate and sulphur compounds), mainly by Deltaproteobacteria and Gammaproteobacteria, which are part of the core microbiome of mangroves worldwide. Desulfobacterales (anaerobics involved in S and C cycling, and methane and nitrogen transformation) was the most abundant order, and Euryarcheota (Archaea) (active carbon transformation through methanogenesis) is the second most abundant group. Other ubiquitous phyla comprise Planctomycetes, Acidobacteria, Bacteroidetes and Chloroflexi. This microbiome diversity (Shannon Index) shows a significantly positive correlation with salinity, organic C, potential evapotranspiration, minimum temperatures year-round and a significantly negative correlation with annual precipitation. These responses to environmental parameters suggest adaptation to the typical stressful conditions of the SAEC and favour adaptions to a changing environment, of increasing salinity intrusion and decreasing annual rainfall, such as landward migration and increasing nutrient cycling efficiency (Tavares et al. 2021).

### Bioturbators/burrowers

Several functional roles are performed by semiterrestrial Decapods (Crustacea: Decapoda), which are one of the most ecologically significant macrobenthic organisms. This group is dominated mostly by several Brachyuran crabs, such as grapsoids, ocypodoids and xanthoids. Bioturbation by burrowing is mostly driven by several Sesarmids (Grapsoidea), fiddler crabs and *Ucides cordatus* (Ocypodoidea), and *Panopeus* sp. and *Eurytium limosum* (Xanthoidea) (Ferreira and Sankarankutty 2002; Ferreira et al. 2019b). Ocypodid crabs tend to be richer in species in intermediate latitudes (Teles et al. 2024), but SAEC mangroves have relatively rich Brachyuran communities, mainly of Grapsids.

Mostly are fossorial species that can be found associated in communal anastomosed long-lasting tunnels (except individual-burrower fiddler crabs) between the roots of *R. mangle* (Ferreira et al. 2019b), contributing to oxygenating underground tree roots and adjacent soil, and also used by several juvenile and adult fishes, including cyprinodonts, gobiids, fundulids, rivulins, poeciliids and eleotrids for protection (Barletta et al. 2000; Lewis and Gilmore 2007; Lira et al. 2021). In general, the root system of *Lumnitzera racemosa* and *Avicennia* spp. seems to impair the construction of these multibranched systems, thus decreasing crab diversity (Ferreira et al. 2019b).

Through burrowing and consequent soil bioturbation these ecosystem engineers can also influence the microbiota and infaunal diversity, and through changes in sediment and porewater physical chemistry, they can influence nutrient availability and therefore tree species growth and productivity (Warren and Underwood 1986; Kristensen 2008; Ferreira et al. 2019a; Barbanera et al. 2022). In SAEC, burrowing by some fiddler crabs (Ocypodoidea) can bury small mangrove propagules, like those of *L. racemosa*, promoting the dominance of large *R. mangle* propagules, a tree species with higher biomass and C stock (Ferreira et al. 2019a).

### Herbivores

Herbivory in mangroves is predominantly performed by crustaceans, insects and also gastropods and depends on the chemical characteristics of leaves of tree species. In general, mangrove leaves present chemicals to support resilience to strong ultraviolet radiation and high soil salinity, which also work as deterrents to herbivores. For example, *R. mangle* and *L. racemosa* showed significantly lower area eaten and number of leaves attacked than *Avicennia schaueriana*, which was attributed to leaf chemical composition of this salt-excreting species, with higher content of sodium, crude fibre, ash content and lower content of total phenols and soluble carbohydrates (Lacerda et al. 1986).

Leaf eating crabs are important for energy and carbon flow and provide a food source for predators (Ashton et al. 2003). Crab faeces are rich in nitrogen (Lee 2008) and combined with sloppy feeding (Camilleri 1989) produce smaller fragments available for deposit feeders. One of the most important herbivores at soil level is the big crab *Ucides cordatus.* By removing the leaf litter and storing below-ground in their burrows, carbon is retained within the mangrove system. Conversely, *Aratus pisonii* feed on mangrove leaves in the canopy. This crab shows a preference for the leaves of the red mangrove *R. mangle* over *L. racemosa* and *Avicennia germinans.*

While herbivory has not been reported to affect canopy characteristics in mangroves in the SAEC, it can, through differential propagule consumption and herbivory depending on tree species, determine the tree type that establish/remain in a site, and thus the structural/architectural features and biomass of the forest, which can indirectly influence infaunal diversity and nutrient cycling (Smith et al. 1989; Alongi and Christoffersen 1992; Ferreira et al. 2019a; Barbanera et al. 2022). The neotropical crab *Goniopsis cruentata* has a significant structural role in the forest through the higher consumption of propagules of *L. racemosa* and *Avicennia* sp., thus promoting the predominance of *R. mangle*, which is architecturally more complex and consequently richer in crab species among and over roots’ habitats. Abiotic (tides, rain, temperature, soil) and biotic (FG composition) differences between humid and semiarid mangroves are potentially able to determine differences in tree species composition (Ferreira et al. 2019a).

Studies on functional roles of insects in mangroves of the Brazilian Equatorial Margin are very scarce, limiting our knowledge on their full ecological functions in the community. The extent of herbivory depends on diverse factors affecting the palatability and nutritional value of leaves that vary with age, season and between species. Insect herbivory removes less than 5% of leaf biomass, thus with small impact on C and nutrient cycling. However, some insects can cause heavy defoliation events, despite being limited by tannin content (Lacerda et al. 1986; Hogarth 1999; Cannicci et al. 2008).

Defoliation by insect and consumption of apical buds, despite, in general, not deadly to the trees, can potentially reduce reproductive and vegetative growth, reducing reproductive output and hence influencing tree species recruitment, frequently associated with anthropogenic activities impacting on mangroves (Krauss et al. 2008; Lu et al. 2019; Maldonado-López et al. 2019). In the SAEC, insect herbivory was strongly associated with abiotic and biotic factors. Higher intensity of leaf consumption by insects occurred in the dry season, when monthly rainfall varied from 2 to 4 mm and water salinity was >54‰. In the rainy season (70–290 mm; 34–35‰) the degree of total foliar herbivory increased, mostly in *L. racemosa* and *R. mangle* (Silva and Maia 2022)

### Wood borers

Several marine isopods (Crustacea), Teredinidae mollusks (shipworms) (e.g. *Teredo* spp.) and wood-boring coleopterans (e.g. cosmopolitan *Coccotrypes rhizophorae*) are wood borers in mangroves. They can affect the development and even survival of mangrove trees, hence affecting tree diversity and forest architecture (Perry and Brusca 1989; Svavarsson et al. 2002), leading to changes in live and dead biomass and thus aboveground carbon stock. Unfortunately, no study on the effects of marine isopods is known for the SAEC. Teredinids, on the other hand, burrow into mangrove wood and are important in breaking down dead wood, having a significant role in biodegradation, and when abundant can affect the amount of carbon stored in, and released by, the forest. Yet, vacant teredinid tunnels can be exploited by many macro-benthic taxa (e.g. fishes, octopus, polychaetes), enhancing trophic and functional resilience (Hendy et al. 2014, 2022). The impacts of this FG, however, have hardly been studied along the Brazilian Equatorial Margin, the few reports come from the AMMC, where teredinid molluscs are a significant component of local peoples’ traditional diet, and show they display seasonality with higher activity during the wet season (Filho et al. 2008).

## Biogeochemistry

### Biomass, productivity and carbon stocks

Mangroves from the Equatorial Margin of Brazil are comparatively less known in terms of biomass and carbon (C) stocks than their counterparts on the southern coasts. Table 2 summarises the few most complete studies with comparable methodologies that allows an evaluation of differences between biomasses and C stocks of mangroves from the two subregions of the Equatorial coast (AMMC and SAEC). The small number of studies impedes a generalisation of the findings. However, some results are outstanding. SAEC mangroves present lower aerial biomass and aerial C stock by a factor of 2–5 relative to mangroves in the AMMC. This results from poor structural complexity due to a deficiency of freshwater supply, higher salinity and low inputs of continental-derived nutrients. However, they show similar belowground biomass and soil C stock (Table 2). Exceptionally high aboveground biomass (AGB) values and C contents are found in the Parnaíba River Delta, a 3,700 km^2^ mangrove forest at the border between the semiarid northeast and the humid Amazon regions. The uniqueness of this area has been highlighted in previous oceanographic studies (Carvalho et al. 2017; Chielle et al. 2023a, 2023b), but the logistic and methodological challenges have hampered more detailed studies in the region and proper estimates of BGB and soil C stock are not yet available.

**Table 2.** Comparisons of carbon and aboveground (AGB) and belowground biomass (BGB) in t ha^−1^ between humid and semiarid mangroves in the equatorial coast of Brazil

Type	Latitude	Annual rainfall (mm)	AGB (t ha^−1^)	C−AGB (tC ha^−1^)	BGB (t ha^−1^)	Soil carbon (tC ha^−1^)
Humid^1,2,3^	0^o^37′–0^o^ 49′ S	2300	290–451	125–196	12	322
Transition^3,4^	2^o^85′S	1320	517	258**	–	–
Semiarid^3,5^	3^o^30′–5^o^44′	1120	86–153*	40–72*	10–14	341

*Notes*: Only forests dominated by *Rhizophora* were used. *Soil carbon transformed from the original unit to tC ha^−1^.**Transformed from biomass to Carbon values using a 0.47 conversion Factor, following Portela et al. (2020) and Schumacher (2002). 1. Santos et al. (2019); 2. Kauffman et al. (2018a); 3. Rovai et al. (2022); 4. Portela et al. (2020); 5. Kauffman et al. (2018b).

Meng et al. (2017) reported a positive relationship between C stocks in AGB and in BGB of mangroves in China and suggested this relationship could be applicable worldwide and thus used to obtain more accurate estimates of mangrove blue C stocks at regional or global scales. Their review, however, failed to include data from the Equatorial Western Atlantic, and the preliminary results available suggest this relationship does not hold for SAEC mangroves. Therefore, predictions of decline of C stock in AGB and BGB (C in roots and soil) under any future climate change scenario may result differently depending on the forest type and location (Singh et al. 2022).

Season is an important variable regulating forest productivity in the SAEC, being higher in the rainy season, much like any other mangroves worldwide (Portela et al. 2020; Gomes et al. 2021). Highest productivity occurs under low soil and porewater salinity and with adequate supply of nutrients and freshwater restricted to the short wet season. In pristine mangroves in the SAEC, increased fluvial discharge strongly influences nutrient concentrations and therefore availability to mangroves. Higher dissolved N and soluble reactive P concentrations occurs in the rainy season (Barroso et al. 2016; Silva et al. 2009; Nóbrega et al. 2013), a pattern also observed after storms events in other semiarid coasts of the world and attributed to increased leaching and transport of materials from river upper basins and fluvial waterways (Eyre and Ferguson 2005). This nutrient pulse during the short rainy season may be responsible for up to 85% of the total nutrients exported to the lower estuary. Unfortunately, to our knowledge, there is no estimate of mangrove litterfall rates in SAEC. However, mangrove forests under similar climatic and geological conditions shows litterfall rates in the same range of values observed in semiarid littorals; 82 gC m^−2^ year^−1^ in the Gulf of California, Mexico (Arreola-Lizarraga et al. 2004); 212 gC m^−2^ year^−1^ in Karachi, Pakistan (Farooqui et al. 2012) and from 57 to 238 gC m^−2^ year^−1^ in semiarid Caribbean mangroves (Lacerda 2002). Based on the C content of typical thyolitic gleysols, mangrove soils (Suárez-Abelenda et al. 2014; Nóbrega et al. 2019) estimated extremely high soil C stock of 8,200 ± 900 gC m^−2^. Unfortunately, this extremely high estimate cannot be confirmed by field data. Year-round larger fluxes in the AMMC result in highest litterfall that vary from 51 to 203 gC m^−2^ year^−1^ (Gonçalves et al. 2006; Nascimento et al. 2006; Fernandes et al. 2007; Mehlig 2001).

Average whole ecosystem carbon stocks per unit of area in AMMC mangroves (361–746 t C ha^−1^; at 0°40′S and annual rainfall of 2,300 mm) (Kauffman et al. 2018b); and those from the humid eastern coast, the Jaguaripe estuary in Bahia State (at 13°11′S, 1 and annual rainfall of 350 mm) are similar (250–633 Mg C ha^−1^) (Hatje et al. 2021). This clearly confirms higher carbon stocks are more clearly associated with humid climate, rather than latitude. An exception is the urban-influenced mangroves in the SAEC, where soil carbon sequestration rates, due to high allochthonous aeolian and urban inputs of organic material rather than autochthonous production, are up to 14 times higher than the global average (Ward et al. 2023). A detailed characterisation of the mangrove soils organic matter in urban-impacted and rural areas mangroves found a significant contribution of anthropogenic sources in the total carbon accumulated in sediments, and a clear increase in importance of anthropogenic carbon in the more urbanised sites (Mounier et al. 2018). Passos et al. (2021) reported increasing accumulation rates of total organic carbon and total nitrogen in the Suape estuary, in NE Brazil following the port-industrial facilities development starting in the 1980’s, reflecting urbanisation and industrial growth. The anthropogenic contribution was clearly shown by the observed heavier δ^15^N values in the sediment column. This scenario, however, seems not exclusive of SAEC mangroves, since in humid coastlines, mangroves adjacent to or within metropolitan regions show increased sediment accretion rate (SAR) and carbon sequestration and contents in sediments (Sanders et al. 2014). Robust sampling undertaken along estuarine gradients strongly suggests that soil Corg stocks are considerably less variable along the sea–land estuarine gradient than across the intertidal gradient from the water edge to the border with terrestrial vegetation. Although AGB is much more variable along the longitudinal estuarine gradient, the highest AGB is observed in the lower estuary and lowest ABG in the upper estuary (Hatje et al. 2021).

### Sulphur and iron soil chemistry

The AMMC is dominated by soils with significant amounts of Fe and Al, classified as Ferri-humuluvic Spodosols and Hydromorphic Arenics, a few meters in thickness, with horizons containing significant proportions of organic matter and Fe. Mangrove soils, mostly halomorphic and hydromorphic Gleisols in the AMMC, receive a large amount of Fe from upland soils. Clay and silt fractions predominate with moderate to high amounts of organic matter and soluble salts. They are weakly consolidated, greyish to black in colour, with the overwhelming presence of H_2_S (Schaefer et al. 2017). Iron content is unaffected by seasonality due to a surplus of water even in the dry season. These circumstances promote nearly permanent anoxic conditions and allow the precipitation of pyrites and the accumulation of chalcophile elements, including toxic heavy metals of environmental significance, such as Cd, Pb, Hg, Zn and Cu.

SAEC mangroves soils are characterised by tertiary and quaternary deposits forming coastal plains constituted of sandy soils closer to the coast and yellow-red latosols (mostly oxisols) inland (Lacerda et al. 2008). They are relatively poorer in iron content resulting from the relatively smaller Fe input from upstream basins (Ferreira et al. 2007, 2021). During the wet season, suboxic to anoxic conditions may develop as in the AMMC. A strong water deficit in the dry season and increased flooding by oxic seawater solubilise deposited sulphides and releases Fe and heavy metals to porewaters, which are converted to Fe oxyhydroxides at the rhizosphere level.

**Figure 3.**
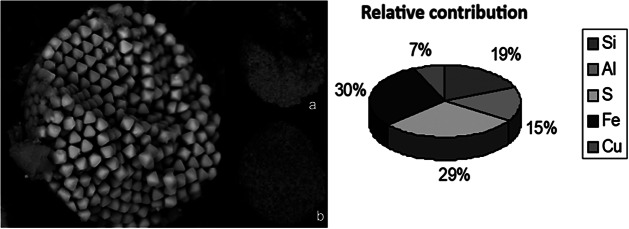
Framboidal pyrite forming in mangrove sediments from the SAEC of Brazil, mostly formed by sulphur (a) and iron (b), but with considerable contents of other elements, including toxic heavy metals.

The production of sulphides derives from the anaerobic decomposition of organic matter. Sulphides accumulate in the sediment porewater and may exceed the tolerance threshold of mangroves. In the AMMC, Fe(III) (hydr)oxides efficiently mitigate sulphide toxicity to mangroves by partially avoiding rapid sulphide accumulation by sequestering it in the sediment in the form of pyrite or jarosite (Cobacho et al. 2024). This adaptation may be impaired during the long dry season in the SAEC resulting in higher toxicity to mangrove plants. Framboidal pyrite crystals about 40 μm abound in the sediments of SAEC mangrove soils, an example is shown in a scanning electron microscopy photograph in Figure 3. The X-rays analysis of this framboid reveals the dominant presence of S (Figure 3a) and Fe (Figure 3b), elements forming the most common type of pyrites. Through the same technique, it is possible to reveal the presence of chalcophile toxic heavy metals in the framboid, such as copper (Cu). These metals can be remobilised to porewaters during the longer dry season.

The strong seasonal shifts of fluvial flux variability in the SAEC strongly affects biogeochemical processes at the soil–air interface, including evapotranspiration that significantly affect redox-sensitive biogeochemical processes, including root radial oxygen loss, iron plaque formation through iron and sulphate reduction and pyrite oxidation. These will impact on productivity, biomass growth and root exudate release, potentially affecting fauna activity, and in the case of FG (Araújo Júnior JM et al. 2016), key biogeochemical processes and ecosystem functioning and services. Precipitation of carbonates may occur, as soil contents can vary from 4 to 11%, contributing to SARs (Albuquerque et al. 2014). Redox-sensitive micronutrients, such as Fe and Mn, are particularly affected by shifts in fluvial fluxes (Lacerda et al. 2022b; Aragon and Miguens 2001) and can eventually impact on nutrient balance and availability of plant uptake as well as export to adjacent coastal areas, in particular phosphorus (Silva et al. 1998; Marins et al. 2020).

## Response to anthropogenic stressors

### Contamination and pollution

Sulphate reduction by-products pose threats to mangroves that adapt via a range of anatomical and physiological mechanisms dependent on species, which eventually control pollutant transfer. Distribution of iron in the rhizosphere of the dominant neotropical mangrove species shows the formation of iron-plaques that are the most effective barriers to trace metal uptake and translocation by mangrove plants (Machado et al. 2005; Cheng et al. 2010). Relatively less reducing environmental conditions in mangrove sediments of the SAEC, as discussed in the previous section on Fe geochemistry, may reduce the formation and significance of iron plaques in fixating toxic metals in the rhizosphere. In the semiarid Jaguaribe River Estuary, iron plaques contribute to an average fraction of the total Cu content in roots varying from 25.8 to 42.7%. Minimum contribution, meaning more Cu being uptake by roots, occurred in less reducing Eh, whereas maximum contribution occurred in the more reducing conditions (Lacerda et al. 2024). In these mangroves, Cu concentrations are 10 times higher than those reported for these species in mangroves from humid mangroves in SE Brazil, where soil redox potential is extremely negative (−316 to −327) (Madi et al. 2015), corroborating that the less reducing conditions of the semiarid mangroves result in higher metal availability for plant uptake. In these humid areas, metal retention in iron plaques is much higher, varying from 62% in *Avicennia schaueriana* to 87% in *R. mangle* (Machado et al. 2005). In addition, Fe accumulation in the salt excretion glands of *A. germinans* from the SAEC suggests that salt excretion can help decrease internal plant concentrations of some toxic metals.

**Figure 4.**
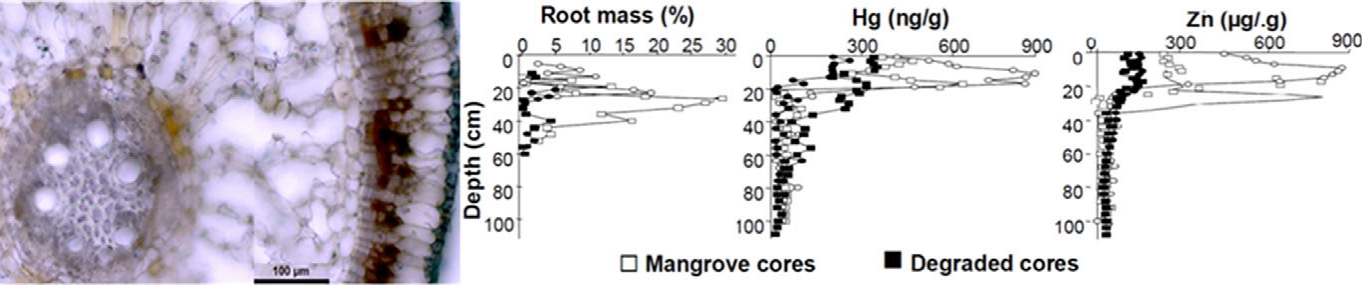
Iron plaque surrounding the external cortex of *R. mangle* roots and root biomass distribution and the distribution of Zn and Hg concentrations in sediment cores from afforested and bare degrade mangroves. Adapted from Lacerda et al. (2024) and Machado et al. (2002), respectively.

The capacity of mangroves to immobilise toxic metals can be used for pollution mitigation measurements and rehabilitation of mangrove as filters to protect adjacent coastal areas from metals leaching from ground water, avoiding contamination of adjacent coastal waters. Figure 4 compares the vertical distribution of selected heavy metals in mangroves from afforested and bare degraded areas sediments surrounding a landfill in a humid region. The strong capacity of mangrove rhizosphere to immobilise metals is clearly shown by the sharp increase in total metal contents at the sediment layers showing highest root biomass in the afforested site, whereas in bare, degraded mangrove sediments metal profiles show a steadily increase in concentrations towards the surface suggest transfer to pore and surface waters. This entire mechanism can be disrupted by the impact on the plant metabolism due to hyper salinity, resulting from reduced rainfall and increased saline intrusion, and smaller porewater contents of dissolved iron due to higher Eh. These are typical conditions found in SAEC mangroves (Lacerda et al. 2022b). This discussion, however, may be very preliminary, due to the scarcity of data on iron plaque formation and metal immobilisation in both the SAEC and AMMC mangroves.

Excess nutrients from anthropogenic sources trigger eutrophication in many mangrove-dominated estuaries along the SAEC, mostly due to poor sanitation, sewage treatment and inadequate waste disposal. This problem may affect the AMMC but considering the extension of their estuaries and coastal mangroves and low density of human population, no studies have quantified the eutrophic state of the region’s mangroves, except for specific locations. In addition, rapidly expanding intensive shrimp farming has greatly increased the eutrophication pressure on mangrove ecosystems in the SEAC due to excess nutrients (Lacerda et al. 2019), and today it is by far the most significant source of nutrients to SAEC mangroves, with the exception of the urban mangroves surrounding the metropolitan areas of northeastern Brazil capital cities. As a comparison, the AMMC has less than 3% of the total operating shrimp farms in the SAEC (Lacerda et al. 2020). Mangroves have been suggested to act as filters to human-derived nutrients, based on actual measurement of a net import of nutrients by mangroves, observations have shown that only a fraction of the nutrient input entering the forest is exported back to adjacent coastal areas (Sanchez-Carrillo et al. 2009; Silva et al. 1998), while others reached the same conclusion by modelling nutrient concentrations in waters as a function of dilution (Bin and Dushof 2004). Therefore, mangroves seem to actively immobilise this element either accumulating them in mangrove biomass and/or sediments. At the SAEC, although nutrient inputs may be limiting, mangroves may attain high productivity, through an efficient recycling of limiting nutrients (Holguín et al. 2001). Sediment fauna also influences this process but seems very site-specific (Ferrante and Fearnside 2019). Marins et al. (2020) and Sanders et al. (2014) reported a continuous increasing threat in total nitrogen and phosphorus, respectively, in mangrove sediments following the increasing intensity of anthropogenic drivers, similar to observations by Queiroz et al. (2020) and extended to a verified increase in GHG emissions (Queiroz et al. 2019; Cotovicz et al. 2021).

### Damming

River damming and waterways diversion causes extensive changes in hydrodynamics and sedimentation in semiarid estuaries, particularly, siltation of estuaries and erosion of the coastline due to reducing sediment supply to the coast (Ward et al. 2023). Most rivers along the SAEC are small and intermittent and show a well-defined hydroperiod (Maltchik and Medeiros 2006). During the dry season, sediments accumulate in the river channels and the freshwater flow is almost non-existent. The highest fluxes in the wet season are capable of transporting large amounts of sediments to the continental shelf. Changes in the drainage of the river basins by flow diversion and the construction of multiple dams in the past three decades (Molisani et al. 2006) simultaneous to the climate change-driven reduction in annual rainfall (Cunha et al. 2019) have reduced freshwater inputs during the rainy season, thereby affecting the fundamental transfer fluxes of water and materials between the continent and the ocean.

The main effects of river damming and diversion are that the reduced and frequently regulated fluxes to estuaries are unable to wash out sediments and those accumulate along fluvial beaches, settle and create new, or enlarge existing islands and bars, creating new space for mangrove colonisation (Godoy et al. 2018). In the Pacoti river estuary, another estuary in the semiarid coast, mangrove expansion occurred over abandoned salt pans and on recently enlarged estuarine beaches and islands, also resulting from decreased and regulated fluvial flow by a sequence of dams built less than 100 km from the mouth of the river, to supply water to the metropolitan region of the Ceará (CE) State capital, Fortaleza. Natural fluxes varying from 1.0 to 19 m^3^ s^−1^ were regulated to 1.7 m^3^ s^−1^ year-round. These new areas were quickly occupied and fixed by mangroves, expanding the forest cover from about 71 ha in 1958 to 142 ha in 1999, following dam construction and further expanding to 144 ha in 2004, probably responding to increased ocean forcing (Lacerda et al. 2007). In addition to controls on river flows, the reduced transport capacity of rivers increased sediment retention in estuaries, which has been aggravated by a simultaneous decrease in rainfall over the SAEC of 4.8–5.6 mm year^−1^ in the last 30 years (Moncunill 2006; Alvalá et al. 2019; Marengo et al. 2018).

### Aquaculture

One of the most significant drivers of environmental impacts on the SAEC mangroves is intensive shrimp farming, mostly after recent changes to the Brazilian Forest Code, that have weakened protection for mangroves and associated salt flats (Ferreira and Lacerda 2016a, 2016b). Although the SAEC has only 4% of the total Brazilian mangrove area (ICMBio 2018), it produces over 96% of cultivated shrimp in the country. While only a small area of Northeast mangroves has been directly converted to aquaculture ponds (ICMBio 2018), this direct and indirect forest loss, reported less than 8% of the total mangrove area of the SAEC (Maia et al. 2006), may be proportionally more significant than in mangroves of the humid sector of the Equatorial coast. The reason for this being that SAEC coastal waters are highly oligotrophic and, therefore, primary productivity and fisheries are largely dependent on nutrient fluxes from mangrove-dominated estuaries.

**Figure 5.**
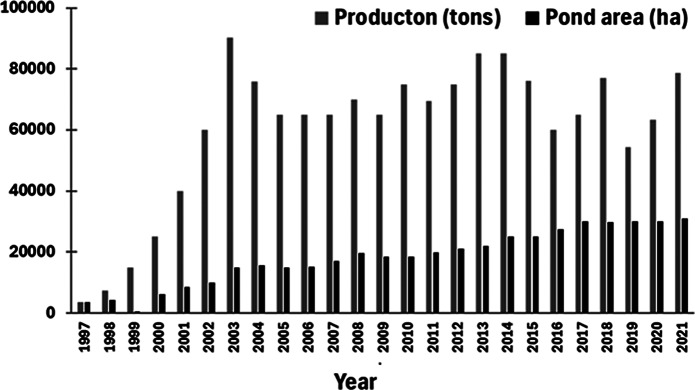
Shrimp aquaculture production and pond area from 1997 to 2021 in the SAEC (adapted and based on figures from Valenti et al. 2021).

Regional intensive shrimp aquaculture in the SAEC, although a relatively recent phenomena, has grown over 2,000% since 1997 (Figure 5), although it has stabilised to an annual production of about 70,000 tons and covering a total pond area of 30,000 ha over the past decade (Valenti et al. 2021). Typically, shrimp ponds are built in mangrove-adjacent salt flats rather than the mangroves themselves, but they maintain periodic hydrological connectivity through dammed channels, allowing the flushing of effluents to local mangrove tidal creeks. The main impacts on mangroves are, therefore, mostly indirect, due to the release of nutrient-rich, oxygen demanding effluents and changes in hydrology, which strongly affect ecosystem functioning, decrease of ecosystem service provision, reduction in nutrients, primary productivity and carbon storage capacity, and the mangrove’s efficiency as an estuarine filter (Lacerda et al. 2021).

Shrimp pond effluents are enriched not only in nutrients but also in trace elements, such as Cu and mercury (Hg) present in aquafeed and chemicals used in the production process. Emission factors of N, P, Cu and Hg are higher than all other anthropogenic sources and concentrations in excess of natural levels and ubiquitous in adjacent tidal creek waters. Mangroves within the Jaguaribe river estuary, a significant production area in the SAEC, with over 3,600 ha of shrimp ponds, have increased annual P emissions by 30% to 43.9 tons, following shrimp pond area increase between 2001 and 2006 (Marins et al. 2011). This was followed by an additional increase to 69 tons in 2013 resulting from another increase in shrimp pond area (Marins et al. 2020), effluents from the local shrimp aquaculture represent over 60% of the total phosphorus load from natural and anthropogenic sources to the lower Jaguaribe Basin (Lacerda et al. 2021). Local mangroves receiving these effluents had their efficiency to accumulate P reduced by over 50%, relative to mangroves in estuaries not affected by shrimp farm effluents, triggering algal blooms and eutrophication in the adjacent estuarine waters (Marins et al. 2020).

**Figure 6.**
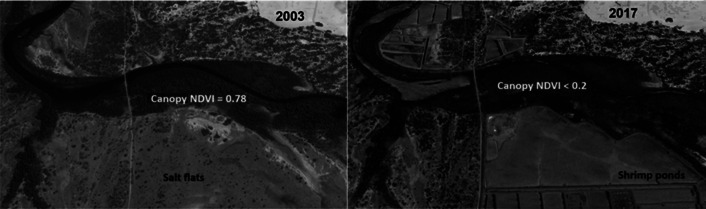
Normalized Difference Vegetation Index (NDVI) of mangrove forests in a Jaguaribe estuary tidal creek receiving shrimp pond effluents in the SAEC.

Changes in tidal creek hydrology also exert a significant impact on mangroves. Reduction in mangrove canopy health adjacent to shrimp farms has been reported and in certain areas has led to the complete degradation and loss of mangrove forests. Normalized Difference Vegetation Index (NDVI) comparing the photosynthetic activity related to canopy structure showed a spatial relationship between mangroves loss and increasing shrimp farm area (Alatorre et al. 2016). At the Jaguaribe estuary, nearly 30% of the total mangrove forest exhibited canopy degradation evidenced by a decreasing NDVI following shrimp farm expansion from 2003 to 2017 (Figure 6). There was a 15% reduction in NDVI between 2003 (0.78) and 2008 (0.65), following shrimp pond area increase from 340 to 1,600 ha; in 2017, there was a further decrease to 0.2, when shrimp farms area increased 10-fold to about 3,400 ha, notwithstanding no direct conversion of mangroves to shrimp ponds. This reduction in the health of mangrove forests is not added to the direct area loss (8%), when computing total area loss of mangrove forest due to shrimp aquaculture. As in the Jaguaribe estuary, this additional canopy loss of integrity would increase the actual mangrove loss up to 15% (Lacerda et al. 2021).

Decommissioning of shrimp farms is not included in Brazilian environmental legislation; soil damage and remaining infrastructure may impair or complicate mangrove restoration and their long-term existence may trigger the occupation of abandoned farms by other activities, resulting in permanent exclusion of mangroves from these areas (Ferreira and Lacerda 2016a).

### Global climate change

Climate change represents an increasing direct threat to semiarid mangroves (Alongi 2022), whereas indirectly it can reinforce impacts from local anthropogenic activities (Gilman et al. 2008; Moomaw et al. 2018; Ashton 2022). Contrary to mangroves in humid regions, SAEC mangroves are already under stress from natural drivers, and as in other extreme environments, the impacts from climate change are a reality and have already altered mangrove forest’s structure, extent, distribution and functioning along this coast. Impacts of climate change are triggered, mostly from rising temperatures, sea level rise (SLR), coastal acidification, changes in precipitation patterns, increased storms and extreme weather events and rising atmospheric CO_2_, among others (Ward et al. 2016)_._

While occurring and predicted, effects of climate change over mangrove forests have been extensively addressed (e.g. Ward et al. 2016; Ward and Lacerda 2021; Alongi 2022), empirical evidence is still scarce and their indirect effects through impacts on the associated biota are not yet well understood. There are concerns that climate change stressors combined with other anthropogenic stressors impact key biotic FGs resulting in functional degradation potentially eroding resilience and leading to stand dieback (Ferreira et al. 2023) and under certain circumstances, even complete loss of mangroves from certain regions of the SAEC. In addition, the increasing water demand by a growing population along the SAEC requires more damming of rivers and reservoir construction, which will further decrease water and sediment load from the continent to the sea, exacerbating the impacts of climate change.

In summary, mangrove responses to climate change are different, or at least not the same intensity when comparing in semiarid (SAEC) with humid (AMMC) mangroves. For most drivers, impacts are stronger on SAEC mangroves than at the AMMC, mostly due to the already stressful conditions of the SAEC compared to the relatively optimal environmental conditions verified in the AMMC. Table 3 summarises the main climate change impacts on the function of SAEC mangroves from drivers associated with climate change and makes a comparison with humid regions mangroves. Further, each major driver is discussed individually.

**Table 3.** A summary of reported drivers of impacts on mangrove ecosystem functioning associated with global climate change in humid and semiarid coasts of the Equatorial Margin of Brazil

Driver	Impacts on the ecosystem	Humid	Semiarid
Sea level rise ^1,2,3,4,5^	Erosion at the sea margin, tree felling	Intermediate	Major
	Increasing tidal forcing	Intermediate	Major
	Saline intrusion and salinization of porewater	Minor	Major
	Landward migration of mangroves and substitution of seasonal saltmarsh communities	Intermediate	Major
	Remobilization and oxidation of bottom sediments and accumulated pollutants	Minor	Major
Rainfall reduction ^6,7,8,9,10,11^	Reduction of continental runoff, exacerbating the effects of sea level rise leading to hypersalinity, increase sedimentation and residence time of waters in estuaries, favouring reactivity of nutrients and pollutants Increasing diversity of soil microbiota	Minor	Major
Increasing the frequency of extreme events ^8,9,12,13^	Extreme droughts cause higher sedimentation and increase water residence time in estuaries	Minor	Major
	Floods increase export of suspended particles to the continental shelf	Minor	Intermediate
Buildup of atmospheric CO_2_^14^	Increasing forest productivity and litterfall production	Major	Minor
	Fuelling microbial metabolism, including sulphate reducing bacteria	Major	Minor
	Intensify the formation of iron plaques in the rhizosphere	Major	Minor
Acidification^15,16^	Dissolution of carbonates, increase elements solubility, decrease buffer capacity	Minor	Major

*Notes:* 1. Alongi (2015); 2. Ward and Lacerda (2021); 3. Jennerjahn et al. (2017); 4. Makowski and Finkl (2018); 5. Soares et al. (2021); 6. Azevedo et al. (2018); 7. Lacerda and Miguens (2011)); 8. Nguyen et al. (2020); 9. Lacerda et al. (2020); 10. Lei et al. (2019); 11. Bergamaschi et al. (2012); 12. Morgado et al. (2021); 13. Cai et al. (2023); 14. Tavares et al. (2021); 15. Sippo et al. (2016); 16. Borges et al. (2003).

#### Global and regional increases in air and soil temperature

In the recent extreme drought in the Amazon region, temperatures approached the survival threshold of a range of species of fishes, crabs, trees and microorganisms of the Amazon forest (Pörtner et al. 2023). Temperatures at the SAEC under natural semiarid conditions added to global warming may have already been very close to the survival threshold of mangrove fauna and flora. Rising temperatures can affect mangrove sediment microorganism communities that mediate OM composition and nutrient/pollutant availability, and eventually biogeochemical processes (Kristensen 2008; Booth et al. 2019; Fusi et al. 2022). Tavares et al. (2021), demonstrated that humid and semiarid mangroves react differently to increasing temperature. The large buffering capacity of humid mangroves, due to greater freshwater flux and larger and more structured forests, will reduce abrupt changes in temperature. Under semiarid conditions, adaptation to these swift changes in temperature of the already stressed mangroves close to the limits of their autecological tolerances will probably decrease microbiome biodiversity and interrelationships.

Increases in temperature and ocean heat waves in the SAEC impact already stressed invertebrate fauna, crabs in particular, and can increase thermohaline stress over gills, prompting burrowing for protection in some species, potentially altering propagule consumption and recruitment patterns (Ferreira et al. 2015). Temperature increases and greater intensity and duration of heatwaves reduce larval survival recruitment of the fiddler crab *Leptuca thayeri* (Marochi et al. 2022). Effects of increased temperatures can also affect burrowing crabs indirectly through disease outbreaks, algal blooms, eutrophication or hypoxia in mangroves of the SAEC (Orélis-Ribeiro et al. 2011). On the other hand, the dominant anemophily of mangrove tree species and their less specialised association with few unspecialised pollinator insects (Nadia and Machado 2014; Diniz et al. 2022), probably dampens the risk of disruption of pollination function by increasing temperature.

#### SLR

Among many impacts from global climate changes affecting the semiarid mangroves of Brazil, SLR, caused ultimately by ocean warming, results in increasing frequency and intensity of the impacts of marine hydrological events, such as waves and tidal forcing. But even earlier than these catastrophic events, SLR strongly alters hydrology, surface and groundwater salinity and soil stability, challenging mangroves with new environmental situations and competitive requirements (Jennerjahn et al. 2017). In Brazil, mangroves advancing over higher coastal plain vegetation have been recorded, since the last decade of the 19th century probably due to the increase in SLR since the end of the Little Ice Age, with a significant intensification from the mid-20th century onwards (Bozi et al. 2021).

SLR is threatening mangrove ecosystems throughout the semiarid region and is further exacerbated by decreasing annual rainfall and damming of rivers. Along the northern extreme of the AMMC, a study of 38 years of spatial monitoring using Landsat images showed a consistent landward migration of mangroves along the shoreline and at the upper region of estuaries, totalling nearly 160 km^2^ in net area increase (Visschers et al. 2022). At the SAEC, historical series of remote sensing maps showed a consistent mangrove expansion associated with increased sedimentation (Ward et al. 2023) and recolonisation of abandoned salt production ponds and decommissioned shrimp aquaculture farms (Lacerda et al. 2007).

The erosion of fringe forests, the major impact of SLR, is triggered when SLR is greater than the SAR (see example in Figure 2c.1). SAR includes sediment build-up by trapping continental runoff and marine suspended particles and carbonate precipitation. In the SEAC mangroves, SAR is quite variable and range from 1.5 to 2.2 mm year^−1^ in mangroves in rural estuaries to relatively high SAR (3.1–7.6 mm year^−1^) in mangroves thriving along urbanised estuaries (Table 4) (Passos et al. 2021; Ward et al. 2023). Along drier coastlines, such as along the Persian Gulf, the gap between SLR and SAR can be even larger since average SAR in the local mangroves vary little and reaches only 0.21 ± 0.09 mm year^−1^ (Saderne et al. 2018). Along the AMMC, SARs vary greatly between 0.7 and 7.1 mm year^−1^, excluding SEAC urban mangroves, the average AMMC SAR are slightly higher (Table 4).

**Table 4.** Sediment accretion rates derived from ^210^Pb dating (mm year^−1^) and sea level rise data derived from Ward et al. (2023) for the SAEC and from PBMC (2017) for the AMMC

State (region)	Sites	Mean sediment accretion rate (mm year^−1^)	Sea level rise (mm year^−1^)	Local sea level rise (mm year^−1^)
Ceara (SAEC)	Ceará LM	1.9	3.5	1.6
Ceara (SAEC)	Ceará UM	2.2	3.5	1.3
Ceara (SAEC)	Cocó LM	7.1	3.5	–3.6
Ceara (SAEC)	Cocó UM	3.1	3.5	0.4
Ceara (SAEC)	Pacoti LM	2.6	3.5	0.9
Ceara (SAEC)	Pacoti UM	1.5	3.5	2.0
Para (AMMC)	Afua LM	1.4	4	2.6
Para (AMMC)	Afua UM	2.4	4	1.6
Para (AMMC)	Breves LM	3.4	4	0.6
Para (AMMC)	Breves UM	2.2	4	1.8
Para (AMMC)	Camara LM	1.4	4	2.6
Para (AMMC)	Camara UM	2.3	4	1.7
Para (AMMC)	Chaves LM	2.4	4	1.6
Para (AMMC)	Chaves UM	3.8	4	0.2
Para (AMMC)	Jaranduba LM	2.3	4	1.7
Para (AMMC)	Jaranduba UM	7.1	4	–3.1
Para (AMMC)	Pesqueira LM	1.7	4	2.3
Para (AMMC)	Pesqueira UM	0.7	4	3.3
Para (AMMC)	Ponta de Pedras LM	3.1	4	0.9
Para (AMMC)	Ponta de Pedras UM	3.5	4	0.5
Para (AMMC)	São Sebastião LM	3	4	1.0
Para (AMMC)	São Sebastião UM	3.8	4	0.2

*Notes:* LM denotes cores taken from lower elevation mangroves and UM from higher elevation (less frequently inundated mangroves).

Reported SAR values suggests that mangroves, free of other constraints, will eventually adapt to SLR by migrating inland, as observed in different arid and semiarid coastlines, with SLR higher or similar to SAR. Dated sediment cores ranging in extension from decades to millennia provide insightful templates of mangrove response to this pressure along the semiarid coast.

**Figure 7.**
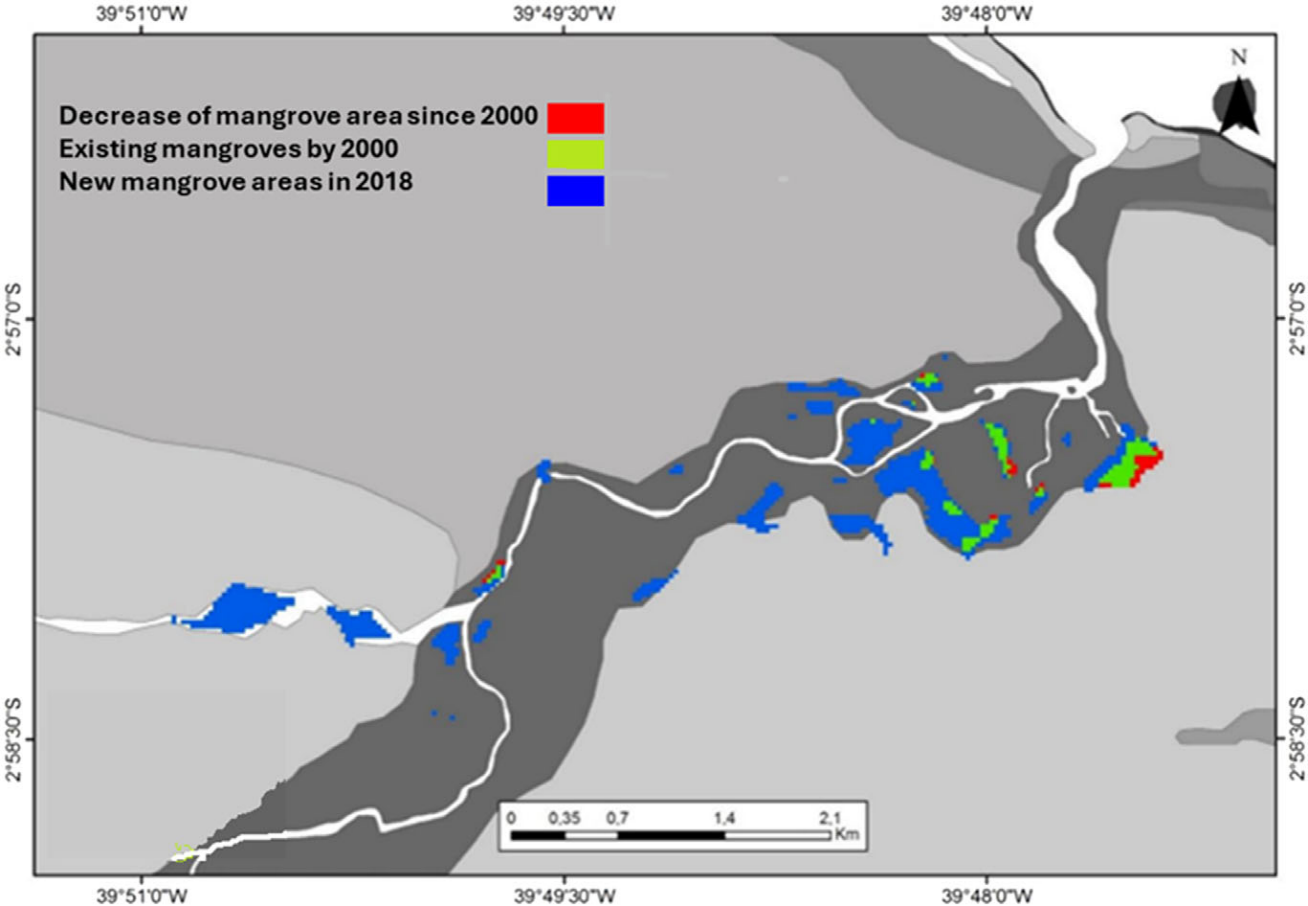
Landward migration of mangroves along the Aracatimirim river estuary, Ceará state in the semiarid northeastern Brazil.

One of the most outstanding mangrove expansions landward of about 400% was estimated at the Aracatimirim River Estuary, also in CE state in the semiarid northeastern Brazil, from the late 1990’s to 2018 (Figure 7). Mangroves occupied sediments in recently formed intertidal islands and enlarged fluvial beaches that were formed following increased choking of tidal estuarine waters by stronger ocean forcing linked to climate change-related heat accumulation in the South Atlantic (Lacerda et al. 2020). Declining terrestrial vegetation by tree mortality as a response to increasing groundwater salinity in low elevation areas and partial replacement by *A. germinans* was reported in Sugarloaf Key, Florida, USA (Ross et al. 2009). Along the equatorial margin of northern Brazil, the location of the most extensive continuous stretch of mangroves in the world (Kjerfve and Lacerda 1993); vast pasture lands on low lying islands and river margins have been replaced by mangroves (Souza Filho and Paradella 2003). This landward migration is the most well documented response of mangroves to sea-level rise (see Godoy and Lacerda 2015, for a review). Although observed worldwide, it is consistently more intense along semiarid coasts, associated with lower annual rainfall and fluvial fluxes, for example, NE Brazil (Godoy et al. 2018).

Annual reduction in rainfall, increased duration and frequency of extended droughts and the effects of floods potentialise the impact of SLR on mangroves, although droughts and floods have an episodic nature, their frequency and intensity have increased due to climate change. The SAEC experienced the worst drought ever recorded in Brazilian history between 2011 and 2017 with a 60% deficit in the average accumulated precipitation relative to historical means (Marengo et al. 2020) that resulted in long-term hypersalinity, strongly affecting mangrove productivity. In semiarid estuaries organisms cope differently with hypersalinity, whereas phytoplankton are relatively well-adapted (Barroso et al. 2018), other taxa respond with changes in composition, diversity and biomass, including mangroves during these extended dry periods (Maia et al. 2018; Garcia et al. 2020). Despite being halophytes, mangrove trees are sensitive to abrupt changes in salinity and prolonged periods of abnormally high salinity. In addition, extended droughts can increase the effects of thermal stress in soil organisms, including crabs, which can decrease litter transformation in detritus. While mangrove swamp crabs are expected to be good osmoregulatory organisms (Burggren and McMahon 1988), sudden or permanent changes in porewater salinity can cause mass mortality by osmotic accommodation failure, particularly if synergistically occurring with high temperatures (Nurdiani and Zeng 2007).

Whereas extended droughts directly impact mangrove physiology, extended flooding events, which also tend to increase in frequency following a decreasing number of rainy days and consequently compressing rainfall to shorter periods, can increase anoxia in pore waters affecting iron plaque formation and the nutrient absorption capacity of roots (Kumar and Ramanathan 2015). Prolonged flooding shifts the sediment and porewater conditions in SAEC mangroves and diminishes plant protection from toxic substances, such as sulphides and trace metals.

Extreme weather events such as those that occur during El Niño events, which are predicted to increase in strength (Cai et al. 2023) can lead to mangrove mortality, mainly by the disruption of soil features from abrupt sea level changes and thermohaline stress (Lovelock et al. 2016; Servino et al. 2018; Ferreira et al. 2023). Soil disruption following these swift changes can also kill micro-and macro-biota that influence biogeochemical cycles, leading to mangrove dieback and release of CO_2_ and nitrous oxide to the atmosphere. Damage by extreme storms and tidal bores is more significant on fringe forests but can be mitigated by trees with large stems and roots or similar aboveground heterogeneous complexity such as pneumatophores, decreasing the force of winds and water currents (Dahdouh-Guebas et al. 2005; Kathiresan and Rajendran 2005). Unfortunately, the natural stressful oceanographic conditions of the continental seaward margin of the SAEC impede the development of robust fringe forests and thus are more sensitive than fringe forests in the AMMC. In addition, mature *Rhizophora* spp., the dominant species in fringe forests in the SAEC lack resprouting meristems, adding additional difficulty to after-event regeneration (Baldwin et al. 2001; Villamayor et al. 2016).

**Figure 8.**
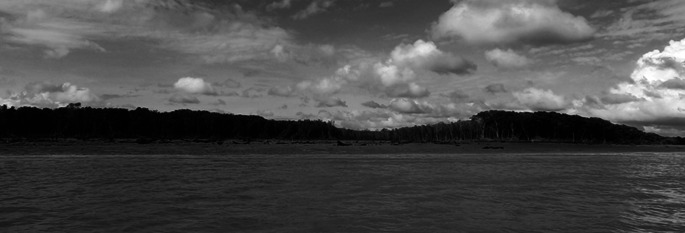
Erosion of large fringing forests dure to extreme flooding and dredging of the estuarine channel in São Luís Bay, at the AMMC.

Erosion of fringe forests (Figure 8) increases suspended solid concentrations in adjacent waters and can locally increase SAR to the level of occluding lenticels diminishing the respiration capacity of mangrove trees and their ability to cope with high salinity. Erosion also accelerates the oxidation of reduced minerals (sulphides) mobilising deposited metals and intensifying the oxidation of sedimentary organic matter, with a resultant increase in CO_2_ emissions.

#### Sand dune encroachment

Mobile dunes are a typical landform of semiarid coasts, and their displacement varies in extent depending on the duration and intensity of the dry season, which in the SEAC depends on the southward migration of the Intertropical Convergence Zone (ICTZ) and the intensity of El Niño Southern Oscillation (ENSO). Since Sea Surface Temperature changes drive the latitudinal position of the ITCZ, there is an intimate relationship between ENSO and the position of the ICTZ. During the dry season, from August to December, when the ICTZ moves northwards, virtually no rain falls (<130 mm) and wind velocity is at its lowest (5.5 m s^−1^). In contrast, precipitation may reach about 1,400 mm and wind velocities can be at their highest average (7.8 m s^−1^). Maia et al. (2005) recorded annual dune displacement in the western coast of the SAEC and observed a relationship with ENSO intensity and duration, with annual average displacement of 17.5 m (14.6–21.0 m) depending on the duration and intensity of the dry season, which is related to ICTZ-ENSO interactions. The estimated associated aeolian transport resulting from these displacement rates averages 102 m^3^ m^−1^ year^−1^ (74–125 m^3^ m^−1^ year^−1^). This seasonal dynamic of mobile dunes is similar worldwide (Abbasi et al. 2019).

**Figure 9.**
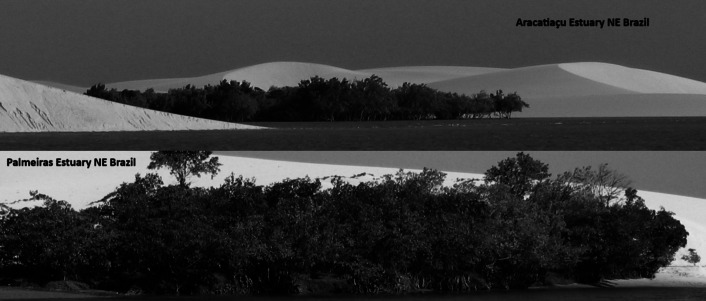
Mobile sand dunes encroaching mangroves in the west cost of Ceará estate in northeastern Brazil.

Mobile dune displacement is accelerating, encroaching adjacent mangroves in the SAEC (Figure 9). Lacerda (2018) argued that this phenomenon is most threatening to mangroves at the interface between the semi-arid and the Amazonian climate, such as the Parnaíba River Delta, that harbours 30,000 ha of mangroves, the largest in NE coast of Brazil. There, larger fluvial fluxes allow glycophytic wetland species, such as *Montrichardia* sp., a typical Amazon basin species, to invade the upper estuary, outcompeting mangroves and impeding their landward migration, exposing them to dune encroachment. In drier conditions, dune displacement over mangroves in the SAEC also favour the invasion of typical dry coastal ecosystems plants species, mostly from the Fabaceae (*Dalbergia ecastaphyllum* L., *Crotalaria retusa* (Forssk.) ‘Schrank’, *Desmodium triflorum* (L.) DC.), Convolvulaceae (*Ipomoea* spp.) and Acanthaceae (*Ruellia paniculata* L.) families, which can compete for nutrients with mangrove species (Lacerda et al. 2022a).

Global changes are causing stronger ENSO associated with a decrease in annual rainfall and increasing frequency and duration of extended droughts over the semiarid region (Marengo et al. 2018; Alvalá et al. 2019), and this has been evidenced by Maia et al. (2005) in dune fields in NE Brazil. Therefore, dune displacement, although of small significance to humid regions mangroves, is becoming a real and present threat to mangrove ecosystems in the SAEC due to climate change.

#### Ocean acidification

A global effect of atmospheric CO_2_ increase is its absorption by the ocean, leading to acidification. Mangrove ecosystems are important carbon sinks but may also act as sources of CO_2_ to the atmosphere. CO_2_ balance and fluxes from mangrove tidal creeks derive from the contribution of mangrove porewater enriched in pCO_2_, dissolved inorganic carbon (DIC) and total alkalinity (TA) and the sediment’s microbial metabolism which produce TA and DIC different anaerobic processes (Chielle et al. 2023a; Sippo et al. 2016). Positive correlations between carbonate system parameters with salinity are significantly higher in the dry season; therefore, in the SAEC, the strong deviation of pCO_2_, TA, and DIC from the conservative mixing curve suggests a significant contribution from organic matter degradation in mangrove-dominated waters. The observed ratio of DIC and TA inputs from SAEC mangroves to coastal waters (Chielle et al. 2023b) results in an overall increase in pH, and thus increasing the buffer capacity of estuarine waters to acidity, an important ecosystem services in a period of global change. The magnitude and extent of this buffering effect is dependent on water residence times and on other sources and sinks of DIC and TA, thus being site specific. The effect of the large TA export from SAEC mangroves and thus their buffering effect on adjacent waters is sensitive to changes in pH and thus could be strongly reduced in a scenario of ocean acidification.

Erosion and tree mortality increase strong oxygenation of sediments, while saline intrusion of ground water brings oxygenated seawater, these processes may induce pyrite oxidation further decreasing pH and increasing acidification. Marins et al. (1997) showed increasing Eh and decreasing pH in sediment porewaters, while Lacerda et al. (2024) showed high heavy metal mobilisation from sediments following seawater intrusion. This mobilisation processes will be enhanced as acidification increases.

## Resilience, recovery and rehabilitation

Mangroves are recognised as resilient shoreline ecosystems over long timescales, including the Holocene fluctuations in sea-level (Alongi 2015), but with different tolerance depending on species (McLeod and Salm 2006). Several natural and anthropogenic degradation drivers, mainly if acting in synergy, can degrade them physically and/or functionally, particularly under the already stressful conditions posed by the semiarid climate. The relatively small area covered by SAEC mangroves relative to the AMMC and other humid areas in the Brazilian coast makes their conservation, and when possible, their rehabilitation/restoration (R/R), extremely important. Protection of existing mangrove stands is mandatory as a first measure to preserve their connectivity and metapopulational structure. As shown in previous sections, SAEC mangroves can colonise ‘apicuns’ (salt flats) or new sedimentary areas created by river damming and/or SLR (Godoy and Lacerda 2015; Ferreira et al. 2022a). They are also able to recover degraded areas if abiotic (mainly soil features and tidal extension) and biotic (interaction with FGs of organisms) conditions are maintained or recovered, assisted or naturally (Ferreira et al. 2015).

Motivated by the decrease in mangrove forest stands at SAEC, many R/R attempts have been made, most of them at low scale, without post-R/R monitoring of recovery attributes and evolution, and occasionally only reported in grey literature. Hence, the area of restored/rehabilitated mangroves at SAEC is unknown. Moreover, the same is valid for mangroves of the AMMC region. SAEC mangrove tree diversity rarely consists of more than three neotropical species in the same stand, so individual species occupy a wider individual niche dimension of the same shoreline functional space respect to more diverse mangroves, such as those in the Indo-West Pacific, facilitating the early succession of a range of native species in R/R projects. In the AMMC region, natural macroscale processes of accretion–erosion of sediment minimise the temporal and spatial magnitude of localised restoration projects, despite the fact that they are valid for specific aims such as stopping localised erosion, recovering fisheries or promoting conservation awareness (Ferreira et al. 2023). There is no published data on medium to large-scale rehabilitation/restoration projects/attempts (RRPA) either in SAEC or AMMC mangroves.

Some small scale or experimental RRPA in the SAEC and AMMC have been published, rendering insights for application in larger areas or to show that mangrove restoration is possible if in the right environmental and social context (Ferreira et al. 2015; Ferreira et al. 2023; Gardunho et al. 2023). In general, assisted mangrove restoration (planting) is only needed if conditions are not able to self-recover, or if a hydrological restoration fails to promote propagule establishment (Lewis 2005, 2009). In the Potengi River estuary, in easternmost SAEC, for example, two cleared adjacent mangrove areas, one (0.67 ha) planted with the original species (*R. mangle*) and the other (2.3 ha) left to self-recover, recovered in few years. The increase in tree biomass was faster in the planted area, but remained monospecific, and seemed reinforced by heavy consumption of *Avicennia* sp. and *L. racemosa* but not R. mangle propagules by Grapsoid crabs, while the self-recovered area took more time to reforest and to reach the high biomass of the former but recovered with the three most common tree species in the estuary: *R. mangle*, *A. germinans* and *L. racemosa.* Studies showed that some significant faunal FGs, like burrower/bioturbator and herbivore/omnivores (mostly consisting in Brachyuran crabs), soon recolonised the areas, first in the planted site, and were thus associated with the dominance of higher-biomass *R. mangle* and consequent higher carbon stock of the forest (Ferreira et al. 2015).

To the west of that site, in the CE State, with a slightly lower average annual rainfall, a 3-ha area of abandoned saltwork was rapidly recovered through hydrological restoration, and the most resistant species to hypersaline soils *A. germinans* and *L. racemosa* (in lower density) were the main colonisers (Ferreira et al., 2022a). In spite of the slow return of FGs (e.g. biogeochemical mediators), ecological interactions like facilitation, herbivory and bioturbation are increasingly shaping the establishing forest. This showed that estuarine salt flats are areas prone to be colonised by mangroves when ongoing SLR push mangroves landward in the SAEC. A nearby 1.75-ha area in the Cocó River mouth was restored by planting *R. mangle*, with planted fragments of 3 and 7 years. Recent studies compared these planted fragments with natural and degraded surrounding areas and found a trend in higher fine sediment and carbon accumulation with mangrove age, with the highest values found in the mature mangrove patch due to higher soil C inputs from root growth and exudates, increased microbial biomass and plant litter (Jimenez et al. 2021), which are typical of *Rhizophora* forests (Ferreira et al. 2019a; Ferreira et al. 2019b). These findings confirm the effectiveness of RRPA to restore soil properties, as well as the high efficiency of *R. mangle* as a key species for neotropical and SAEC mangrove rehabilitation but always allowing the promotion of further establishment of other mangrove species like the recovery capacity over semiarid grounds by *A. germinans.* In addition, while management issues for restoration/rehabilitation of larger mangrove areas in the SAEC await to be tested, a patched restoration applying different techniques appropriated to the state of the targeted fragment or an expected climate effect should be aimed. This may include from passive recovery to a gradient of assisted R/R, a kind of an ‘in-mosaic or patchwork restoration’, that seems to function well for SAEC estuarine mangroves recovery.

In the AMMC, in the State of Pará, several sites covering a total of 14 ha were restored (replanted after clearing by wood extraction) with *R. mangle* and are now dominated by *L. racemosa* and *R. mangle.* The most significant bioturbator/herbivore leaf consumer crab *Ucides cordatus* (an important item in the food and income of the native populations) increased their populations in the recovered areas (which was one of the aims of the RRPA), as well as other ecosystem goods and services that the native inhabitants of the areas helped to restore (de Aviz et al. 2020; Gardunho et al. 2023).

Beyond the direct effects of climate change on the diversity and structure of forests, their indirect effects through organisms (and FGs) directly associated with mangrove functioning can alter the ecological processes of the forest, impairing recovery or leading to further mangrove degradation and decrease of functionality and resilience, and/or mangrove dieback. Forest fragmentation is one of the main drivers of forest degradation, since it decreases ecosystem service provision by mangroves, limits their capacity to resist climate change drivers, allows more human invasions and decreases the continuity of organisms’ populations and their genetic flux (Bryan-Brown et al. 2020). In spite of the size and extent of humid Amazonian mangroves, damage can also be significant, as seen by the ongoing falling of huge trees, in mangrove stand at the margins of Baia de São Marcos, in front of the capital of Maranhão State, São Luiz, driven by channel dredging and SLR.

Through restoration and recovery programs important lessons are to understand the dynamics of targeted areas and the local autecological preferences of the tree species allowing the selection of appropriate R/R strategies (e.g. passive or different degrees of active recovery) (Ferreira et al. 2015, 2022a). Some key faunal (soil microbiota, Brachyuran crabs) and vegetal (red mangrove *R. mangle*, herbaceous halophytes) components are important in functional mangrove community recovery and as indicators of R/R success (Ferreira et al. 2015; Ferreira et al. 2019b; Jimenez et al. 2021). Climate change impacts are posing a challenge to rehabilitate mangroves in the SAEC, especially in areas exacerbated by other hum impacts (Lacerda et al. 2022b; Ferreira et al. 2023).

In addition, R/R programs that have been initiated in areas converted to salt works or shrimp farms are likely suffer from delayed recovery due to soil degradation and impairment of hydrology. Political lobbies are connected to these enterprises, so legal frameworks are constantly backtracked (Ferreira and Lacerda 2016a; Ferreira and Lacerda 2016b; Lacerda et al. 2019, 2021). Extreme levels of OM, Hg and Al in soils with deposition of shrimp ponds effluents have been observed, potentially causing indirect mangrove degradation (Costa et al. 2013; Lacerda et al. 2021).

## Ecosystems services and management

### Ecosystem services provided by mangroves

Notwithstanding the relatively small area of mangroves on the SAEC, they provide critical ecosystem services that underpin environmental health and human well-being. These services include not only coastal protection (Zamboni et al. 2022) and carbon sequestration (Souza et al. 2023) but also cultural services (Queiroz et al. 2017).

The importance of mangroves as natural barriers against storm surges and coastal erosion along the SAEC has been shown to reduce shoreline exposure to coastal hazards and, therefore, helps safeguard population settlements along a mangrove area in Rio Grande do Norte state, at the easternmost sector of the SEAC (Zamboni et al. 2022).

Carbon sequestration is another crucial service provided by these mangroves. Mangroves on the SAEC contribute to carbon storage and sequestration in below- and above-ground biomass (Souza et al. 2023). The possibilities with Blue Carbon in the SEAC have been celebrated under the assumption that promoting blue carbon can be considered an environmentally responsible strategy and a key measure to ensure a sustainable and prosperous future for the region. However, the same study warns that successful implementation requires the collaboration of various stakeholders, including governments, local communities and non-governmental organisations (Tavares et al. 2023).

One ecosystem service that is frequently overlooked is the cultural aspect. Local communities in the northeast region of Brazil have identified four additional cultural services associated with the preservation of traditional ecological knowledge. These include fostering and maintaining social relationships, personal satisfaction and mental and physical relaxation (Queiroz et al. 2017). Local communities have a symbolic relationship with mangrove forests that extends beyond the typical material perspective used to value ecosystem services. This implies that policymakers should consider the socio-cultural dimension of mangrove services a crucial criterion when addressing the major challenges in coastal ecosystem conservation. However, cultural services provided by northeastern mangroves in Brazil are frequently overlooked in policy-making processes. This oversight is likely indicative of a broader global trend where mangrove ecosystems’ cultural and spiritual values are similarly undervalued.

### Conservation status of Brazil’s semiarid mangroves

Despite the vast provision of ecosystem services, mangroves on Brazil’s semi-arid coast are under significant threat, from the deforestation for agriculture and urban development and the pollution from industrial activities to the expansion of shrimp farming (Ferreira and Lacerda 2016a). The detrimental impacts of shrimp farming are particularly concerning, including habitat degradation and water quality deterioration resulting from effluent discharge in northeast Brazil (Lacerda et al. 2021).

In response to these threats, the National Action Plan for the Conservation of Mangroves outlines strategies to protect and restore mangrove areas through legal protections, restoration projects and sustainable management practices (MMA 2015; ICMBio 2019). Another legal instrument protecting mangroves in Brazil is the 2012 Forest Code, which classifies mangroves as permanent protection areas. However, literature on the subject indicates that the 2012 revision of Brazil’s Forest Code has introduced changes that have weakened the protection of mangrove ecosystems (Borges et al. 2017). In present year, a special law (Decree. Nr 12.045/2024) launched in Brazil the ‘*National Program of Conservation and Sustainable Use of Mangroves – ‘ProManguezal’*) to promote the conservation, recovering and sustainable use of Brazilian mangroves.

Against the backdrop of environmental legislation that has been weakened by Congress and governments in Brazil (Soares-Filho et al. 2014; Ferrante and Fearnside 2019; Losekann and Paiva 2024), the National System of Conservation Units (SNUC) – which establishes protected areas and promotes sustainable natural resource use (Brasil 2000; MMA 2015) – is one of the most robust pieces of legislation to protect ecosystems in Brazil.

In Brazil, 87% of the entire mangrove environment is located within protected areas (ICMBio 2018). However, assessing the effectiveness of mangrove conservation within protected areas in the SAEC presents mixed outcomes. Despite the presence of a dedicated manager and considerable community support in a mangrove protected area in CE state, significant improvements are necessary across all management dimensions. None of the dimensions assessed achieved a ‘satisfactory’ or ‘very satisfactory’ rating (Araruna and Soares 2017). The study identified several areas for improvement, including the need to increase human and financial resources, update and refine management plans and expand environmental education initiatives within the communities.

Further research within the mangroves of marine protected areas in CE state indicated that local stakeholders support the protected areas and desire greater engagement from management institutions (Araruna and Soares 2017; Maia et al. 2019), with a need for a more inclusive approach that addresses local needs (Ternes et al. 2023).

Some of these protected areas rely heavily on co-management strategies or some other degree of involvement of local communities in mangrove conservation. In a review of community-based mangrove management worldwide, Datta et al. (2012) emphasise the effectiveness of involving local communities in conservation efforts, which has improved ecosystem health and resilience. However, the equitable distribution of accrued benefits and services among community members is also a significant concern in these initiatives. Community-led governance, which involves considering local knowledge in selecting rehabilitation and management strategies, encourages genuine participation through mutual assistance and enables independent collective decision-making. For example, the creation of the ‘Sustainable Use Reserve’ (RDS) Ponta do Tubarão in RN State was a demand of the native communities, aiming to break the expansion of aquaculture and real estate speculation (Mattos et al. 2011). However, the success of coastal protection is also influenced by geomorphological traits, indicating the need for an integrated strategy that combines physical and social aspects when shaping community participation (Damastuti et al. 2023).

These concerns extend to management instruments on Brazil’s semiarid coast, where conservation-related subsidies have and will continue to impact the living conditions of local populations positively. However, the impacts on ecosystem health are perceived as a potential concern that has not yet been realised (MDSCF 2016). Irrespective of the legal instrument in place, enforcement remains largely inadequate (Ferreira and Lacerda 2016a). Consequently, the continued occurrence of illegal activities threatens mangrove health. Therefore, it is imperative to reinforce implementation and monitoring efforts to ensure the long-term conservation of these ecosystems, particularly along the semiarid coast, where the natural extreme climate conditions, worsened by global warming, highlight their importance to local traditional populations.

### Conservation challenges on the semiarid coast

The specific characteristics of these mangroves have a direct impact on the conservation and management efforts that are undertaken. These characteristics relate to the geomorphological traits of the location of these mangrove areas, the tidal regimes and the nearby environment, which may or may not be adequate for a possible landward expansion of mangroves in a sea-level rise scenario. These traits directly impact the ecological and biological features of these mangroves. A second set of characteristics has to do with the history of the colonisation of the Brazilian northeast coast, which explains the current urbanisation and other land use change patterns that directly affect northeastern mangroves.

Regarding geomorphological and geographical aspects, the available area for mangrove migration is restricted on several sites on the semiarid coast due to urban development expanding at the edge of estuaries (Ward et al. 2023). Additionally, large dune systems along the coastline are natural barriers to mangrove expansion (Lacerda 2018). This may result in mangroves, like other coastal elements, becoming encroached by mobile dunes under the current climate emergency (Maia et al. 2005).

Vis-à-vis historical degradation patterns and poverty on the semi-arid coast, the Brazilian northeast is one of the poorest regions in the country. Therefore, the significance of mangrove ecosystem services is intensified by the pervasive socioeconomic distress experienced by a considerable proportion of the population, including poverty and hunger (Ottonelli and Mariano 2014; Caldas and Sampaio 2015) and insecure employment (Silva Filho and Queiroz 2011). Consequently, the challenges associated with mangrove conservation include the consideration of the impact on local populations, who often depend on these ecosystems for their livelihoods.

## Conclusions

Mangroves within the SAEC region of northeastern Brazil are able to develop and be resilient in a semiarid coastal environment, but mostly human direct and indirect (i.e. climate change) impacts threaten them. These mangroves are of ecological and economic importance and their support to large traditional fisheries and high biodiversity, including some threatened species. They present lower aboveground biomass compared to humid mangroves of the AMMC but show similar belowground biomass and soil carbon stocks. Iron geochemistry is a primary driver of soil characteristics in SAEC mangrove, suggesting different responses to climate change drivers compared to AMMC region mangroves. Notwithstanding legal protection, SAEC mangroves are witnessing progressive degradation due to regional drivers, which differs from those in the AMMC region, mostly aquaculture and river damming, potentialised by global climate change. These conditions occur at a global scale; however, the impacts in the SAEC are worsened by the natural conditions of semiarid coastlines, which already provide biologically stressful conditions for mangroves. The main strategy to conserve ecosystem services from SAEC mangroves is to preserve and expand the remaining forests. However, where assisted recovery, rehabilitation/restoration projects are required, appropriate consideration should be taken concerning species selection in light of local conditions, including anthropogenic pressures and climate change impacts. SAEC mangrove tree diversity rarely comprises more than three species in the same stand, each species occupying a wider ecological niche at the shoreline respect to more diverse mangroves, facilitating rapid development through the selection of native species with a high recovery capacity, for example, *R. mangle* and *A. germinans.* As noted here, while many of SAEC mangroves are protected, the levels of protection can, in practice, be quite weak, and management should be conducted in partnership with local communities, many of whom rely heavily on mangroves for traditional fishing practices, as well as their importance from a spiritual and cultural perspective, which is often unaccounted in policy and management.

## Data Availability

All data are available by contacting the corresponding author.

## References

[r1] Abbasi HR, Opp C, Groll M, Rohipour H and Gohardoust A (2019) Assessment of the distribution and activity of dunes in Iran based on mobility indices and ground data. Aeolian Research 41, 100539. 10.1016/j.aeolia.2019.07.005

[r3] Albuquerque AGBM, Ferreira TO, Nóbrega GN, Romero RE, Souza Júnior VS, Meireles AJA and Otero XL (2014) Soil genesis on hypersaline tidal flats (apicum ecosystem) in a tropical semi-arid estuary (Ceará, Brazil). Soil Research 52, 140–154. 10.1071/SR13179

[r4] Alongi DM (2015) The impact of climate change on mangrove forests. Current Climate Change Reports 1, 30–39. 10.1007/s40641-015-0002-x

[r5] Alongi DM (2021) Macro- and micronutrient cycling and crucial linkages to geochemical processes in mangrove ecosystems. Journal of Marine Science and Engineering 9, 456. 10.3390/jmse9050456

[r6] Alongi DM (2022) Climate change and mangroves. In Thammineni P and Ashton EC (eds.), *Mangroves: Biodiversity, Livelihoods and Conservation.* Das SC. Singapore: Springer, pp. 175–198. 10.1007/978-981-19-0519-3_8

[r7] Alongi DM and Christoffersen P (1992) Benthic infauna and organism-sediment relations in a shallow, tropical coastal area – influence of outwelled mangrove detritus and physical disturbance. Marine Ecology Progress Series 81, 229–245. https://www.jstor.org/stable/24827336

[r8] Alvalá RCS, Cunha AP, Brito SSB, Seluchi ME, Marengo JA and Moraes OLL (2019) Drought monitoring in the Brazilian Semiarid region. Anais da Academia Brasileira de Ciências 91, e20170209. 10.1590/0001-3765201720170209 29044320

[r9] Andreote FD, Jiménez DJ, Chaves D, Dias ACF, Luvizotto DM, Dini-Andreote F, Fasanella CC, Lopez MV, Baena S, Taketani RG and de Melo IS (2012) The microbiome of Brazilian mangrove sediments as revealed by metagenomics. PLoS One 7, e38600. 10.1371/journal.pone.0038600 22737213 PMC3380894

[r220] Alatorre LC, Sanchez-Carrillo S, Miramontes-Beltr S, Medina RJ, Torres-Olave ME, Bravo LC, et al. (2016) Temporal changes of NDVI for qualitative environmental assessment of mangroves: Shrimp farming impact on the health decline of the arid mangroves in the Gulf of California (1990-2010). J. Arid Environ. 125, 98–109.

[r10] Aragon GT, Miguens FC (2001) Microscopic analysis of pyrite in the sediments of two Brazilian mangrove ecosystems. Geo-Marine Letters 21, 157–161. 10.1007/s003670100078

[r11] Araruna RPL and Soares MDO (2017) Efetividade de manejo em unidade de conservação com manguezais: estudo de caso no litoral do Ceará, nordeste do Brasil. Geosaberes 8, 53. 10.26895/geosaberes.v8i16.597

[r12] Araújo Júnior JM de C, Ferreira TO, Suarez-Abelenda M, Nóbrega GN, Albuquerque AGBM, Bezerra A de C and Otero XL (2016) The role of bioturbation by *Ucides cordatus* crab in the fractionation and bioavailability of trace metals in tropical semiarid mangroves. Marine Pollution Bulletin 111, 194–202. 10.1016/j.marpolbul.2016.07.011 27422484

[r13] Arreola-Lizarraga JA, Flores-Verdugo F and Ortega-Rubio A (2004) Structure and litterfall of an arid mangrove stand on the Gulf of California, Mexico. Aquatic Botany 79, 137–143. 10.1016/j.aquabot.2004.01.012

[r14] Ashton EC (2022) Threats to mangroves and conservation strategies. In Thammineni P and Ashton EC (eds.), *Mangroves: Biodiversity, Livelihoods and Conservation;* Das SC. Singapore: Springer, pp. 217–230. 10.1007/978-981-19-0519-3_10

[r15] Ashton EC, Macintosh DJ and Hogarth PJ (2003) A baseline study of the diversity and community ecology of crab and molluscan macrofauna in the Sematan mangrove forest, Sarawak, Malaysia. Journal of Tropical Ecology 19, 127–142. 10.1017/S0266467403003158

[r16] Azevedo LS, Pestana IA, Rocha ARM, Meneguelli-Souza AC, Lima CAI, Almeida MG and Souza CMM (2018) Drought promotes increases in total mercury and methylmercury concentrations in fish from the lower Paraíba do Sul river, southeastern Brazil. Chemosphere 202, 483–490. 10.1016/j.chemosphere.2018.03.059 29579683

[r17] Baldwin A, Egnotovich M, Ford M and Platt W (2001) Regeneration in fringe mangrove forests damaged by Hurricane Andrew. Plant Ecology 157, 151–164. 10.1023/A:1013941304875

[r18] Barbanera A, Markesteijn L, Kairo J, Juma JA, Karythis S and Skov MW (2022) Functional responses of mangrove fauna to forest degradation. Marine and Freshwater Research 73, 762–773. 10.1071/MF21257

[r19] Barletta M, Saint-Paul U, Barletta-Bergan A, Ekau W and Schories D (2000) Spatial and temporal distribution of Myrophis punctatus (Ophichtidae) and associated fish fauna, in a north Brazilian intertidal mangrove forest. Hydrobiologia 426, 65–74. 10.1023/A:1003939000270

[r20] Barroso HS, Becke H and Melo VMM (2016) Influence of river discharge on phytoplankton structure and nutrient concentrations in four tropical semiarid estuaries. Brazilian Journal of Oceanography 64, 37–48. 10.1590/S1679-87592016101406401

[r21] Barroso HS, Tavares TCL, Soares MO, Garcia TM, Rozendo B, Vieira ASC, Viana PB, Pontes TM, Ferreira TJT, Pereira Filho J, Schettini CAF and Santaella ST (2018) Intra-annual variability of phytoplankton biomass and nutrients in a tropical estuary during a severe drought. Estuarine, Coastal and Shelf Science 213, 283–293. 10.1016/j.ecss.2018.08.023

[r22] Bashan Y and Holguín G (2002) Plant growth-promoting bacteria: a potential tool for arid mangrove reforestation. Trees 16, 159–166. 10.1007/s00468-001-0152-4

[r23] Bergamaschi BA, Krabbenhoft DP, Aiken GR, Patino E, Rumbold DG and Orem WH (2012) Tidally driven export of dissolved organic carbon, total mercury, and methylmercury from a mangrove dominated estuary. Environmental Science & Technology 46, 1371–1378. 10.1021/es2029137 22206226 PMC3280730

[r24] Bernini E and Lage-Pinto F (2021) Infestation of the invasive exotic moth *Hyblaea puera* (Lepidoptera: Hyblaeidae) in *Avicennia* L. (Acanthaceae) in the mangrove of the Mamanguape River, Paraíba, Brazil. Aquatic Botany 168, 103311 10.1016/j.aquabot.2020.103311

[r25] Bin BB and Dushof J (2004) Mangrove filtration of anthropogenic nutrients in the Rio Coco Solo, Panama. Management of Environmental Quality: An International Journal 15, 131–142. 10.1108/14777830410523071

[r26] Blondel J (2003) Guilds or functional groups: does it matter? Oikos 100, 223–231. 10.1034/j.1600-0706.2003.12152.x

[r27] Booth JM, Fusi M, Marasco R, Mbobo T and Daffonchio D (2019) Fiddler crab bioturbation determines consistent changes in bacterial communities across contrasting environmental conditions. Scientific Reports 9, 3749. 10.1038/s41598-019-40315-0 30842580 PMC6403291

[r28] Borges AV, Djenidi S, Lacroix G, Théate J, Delille B and Frankignoulle M (2003) Atmospheric CO_2_ flux from mangrove surrounding waters. Geophysical Research Letters 30, 1558. 10.1029/2003GL017143

[r29] Borges R, Ferreira AC and Lacerda LD (2017) Systematic planning and ecosystem-based management as strategies to reconcile mangrove conservation with resource use. Frontiers in Marine Science 4, 353. 10.3389/fmars.2017.00353

[r30] Bosire JO, Kairo JG, Kazungu J, Koedam N and Dahdouh-Guebas F (2005) Predation on propagules regulates regeneration in a high-density reforested mangrove plantation. Marine Ecology Progress Series 299, 149–155.

[r31] Bozi BS, Figueiredo BL, Rodrigues E, Cohen MCL, Pessenda LCR, Alves EEN, de Souza AV, Bendassolli JA, Macario K, Azevedo P and Culligan N (2021) Impacts of sea-level changes on mangroves from southeastern Brazil during the Holocene and Anthropocene using a multi-proxy approach. Geomorphology 390, 405. 10.1016/j.geomorph.2021.107860

[r32] BPBES (2023) Plataforma Brasileira de Biodiversidade e Serviços Ecossistêmicos (BPBES). *Sumário para Tomadores de Decisão do 1° Diagnóstico Brasileiro Marinho-Costeiro sobre Biodiversidade e Serviços Ecossistêmicos.* Editora Cubo, São Carlos, p. 32. Available at https://www.bpbes.net.br/documentos/STD_MarinhoCosteiro2023-TELA.pdf

[r33] Brasil (2000) *Sistema Nacional de Unidades de Conservação da Natureza (SNUC).* Lei No. 9.985, de 18 de Julho de 2000. Ministério do Meio Ambiente, Brasília, DF. Available at https://www.planalto.gov.br/ccivil_03/LEIS/L9985.htm

[r34] Brown DR, Marotta H, Peixoto RB, Enrich-Prast A, Barroso GC, Soares MLG, Machado W, Pérez A, Smoak JM, Sanders LM, Conrad S, Sippo JZ, Santos IR, Maher D and Sanders CJ (2021b) Hypersaline tidal flats as important “blue carbon” systems: a case study from three ecosystems, Biogeosciences 18, 2527–2538. 10.5194/bg-18-2527-2021

[r35] Brown DR, Marotta H, Peixoto RB, Enrich-Prast A, Barroso GC, Soares MLG, Machado W, Pérez A, Smoak JM, Sanders LM, Conrad S, Sippo JZ, Santos IR, Maher DT and Sanders CJ (2021a) Hypersaline tidal flats as important “blue carbon” systems: a case study from three ecosystems, Biogeosciences 18, 2527–2538. 10.5194/bg-18-2527-2021

[r36] Bryan-Brown DN, Connolly RM, Richards DR, Adame F, Friess DA and Brown CJ (2020) Global trends in mangrove forest fragmentation. Scientific Reports 10,7117. 10.1038/s41598-020-63880-1 32346000 PMC7188678

[r37] Bunting P, Rosenqvist A, Lucas RM, Rebelo L-M, Hilarides L, Thomas N, Hardy A, Itoh T, Shimada M and Finlayson CM (2018) The global mangrove watch – a new 2010 global baseline of mangrove extent. Remote Sensing 10, 1669. 10.3390/rs10101669

[r38] Burggren W and McMahon B (1988) Biology of the Land Crabs. Cambridge: Cambridge University Press. 10.1017/CBO9780511753428

[r39] Cai W, Ng B, Geng T, Jia F, Wu L, Wang G, Liu Y, Gan B Yang K, Santoso A, Lin X, Li Z, Liu Y, Yang Y, Ji F-F, Collins M and McPhaden M(2023) Anthropogenic impacts on twentieth-century ENSO variability changes. Nature Reviews Earth & Environment 4, 407–418. 10.1038/s43017-023-00427-8

[r40] Caldas RM and Sampaio YSB (2015) Pobreza no nordeste brasileiro: uma análise multidimensional. Reviews of Economics Contemporary 19, 74–96. 10.1590/198055271914

[r41] Calderon DG and Echeverri BR (1997) Obtaining *Rhizophora mangle* seedlings by stimulation of adventitious roots using an air-layering technique. In Lacerda LD, Diop S, and Kjerfve B (ed.), Mangrove Ecosystem Studies in Latin America and Africa. Paris: UNESCO, pp. 98–107.

[r42] Camilleri J (1989) Leaf choice by crustaceans in a mangrove forest in Queensland. Marine Biology 102, 453–459. 10.1007/BF00438346

[r43] Cannicci S, Burrows B, Fratini S, Smith III TJ, Ofenberg J and Dahdouh-Guebas F (2008) Faunal impact on vegetation structure and ecosystem function in mangrove forests: a review. Aquatic Botany 89, 186–200. 10.1016/j.aquabot.2008.01.009

[r44] Cannicci S, Lee SY, Bravo H, Cantera-Kintz JR, Dahdouh-Guebas F, Fratini S, Fusi M, Jimenez PJ, Nordhaus I, Porri F and Diele K (2021) A functional analysis reveals extremely low redundancy in global mangrove invertebrate fauna. PNAS 118, e2016913118. 10.1073/pnas.2016913118 34312251 PMC8364210

[r45] Carvalho ACO, Marins RV, Dias FJS, Rezende CE, Lefèvre N, Cavalcante MS and Eschrique AS (2017) Air-sea CO2 fluxes for the Brazilian northeast continental shelf in a climatic transition region. Journal of Marine Systems 173, 70–80. 10.1016/j.jmarsys.2017.02.013

[r46] Cavalcante MS, Marins RV, Dias FJS and Rezende CE (2021) Assessment of carbon fluxes to coastal area during persistent drought conditions. Regional Studies in Marine Science 47, 101934. 10.1016/j.rsma.2021.101934

[r47] Cheng H, Liu Y, Tam NFY, Wang X, Li SY, Chen GZ et al. (2010) The role of radial oxygen loss and root anatomy on zinc uptake and tolerance in mangrove seedlings. Environmental Pollution 158, 1189–1196. 20219275 10.1016/j.envpol.2010.01.025

[r48] Chielle RSA, Marins RV, Cavalcante MS and Cotovicz Jr LC (2023b) Seasonal and spatial variability of CO2 emissions in a large tropical mangrove-dominated delta. Limnology and Oceanography 69, 246–261. 10.1002/lno.12471

[r49] Chielle RSA, Marins RV, Dias FJS, Borges KK and Rezende CE (2023a) Contributions from the main river of the largest open sea delta in the Americas to the CO_2_ fluxes. Regional Studies in Marine Science 62, 102922. 10.1016/j.rsma.2023.102922

[r50] Cobacho SP, Janssen SAR, Brekelmans MACP, van de Leemput IA, Holmgren M and Christianen MJA (2024) High temperature and eutrophication alter biomass allocation of black mangrove (*Avicennia germinans* L.) seedlings. Marine Environmental Research 193, 106291. 10.1016/j.marenvres.2023.106291 38086136

[r222] Costa BGB, Soares TM, Torres RF and Lacerda LD (2013) Mercury distribution in a mangrove tidal creek affected by intensive shrimp farming. Bulletin of Environmental Contamination and Toxicology 90, 537–541. 10.1007/s00128-012-0957-4 23370694

[r51] Cotovicz LC, Ribeiro RP, Régis CR, Bernardes M, Sobrinho R, Vidal LO, Tremmel D, Knoppers BA and Abril G (2021) Greenhouse gas emissions (CO2 and CH4) and inorganic carbon behavior in an urban highly polluted tropical coastal lagoon. Environmental Science and Pollution Research International 28, 38173–38192. 10.1007/s11356-021-13362-2 33723789

[r52] Cunha APMA, Zeri M, Deusdará Leal K, Costa L, Cuartas LA, Marengo JA, Tomasella J, Vieira RM, Barbosa AA, Cunningham C, Cal Garcia JV, Broedel E, Alvalá R and Ribeiro-Neto G (2019) Extreme drought events over Brazil from 2011 to 2019. *Atmosphere* (Basel) 10, 642. 10.3390/atmos10110642

[r53] Dahdouh-Guebas F, Jayatissa LP, Di Nitto D, Bosire JO, Seen DL and Koedam N (2005) How effective were mangroves as a defense against the recent tsunami? Current Biology 15, R443–R447. 10.1016/j.cub.2005.06.008 15964259

[r54] Damastuti E, van Wesenbeeck BK, Leemans R, de Groot RS and Silvius MJ (2023) Effectiveness of community-based mangrove management for coastal protection: a case study from Central Java, Indonesia. Ocean & Coastal Management 238, 106498. 10.1016/j.ocecoaman.2023.106498

[r55] Datta D, Chattopadhyay RN and Guha P (2012) Community based mangrove management: a review on status and sustainability. Journal of Environmental Management 107, 84–95. 10.1016/j.jenvman.2012.04.013 22595074

[r56] de Aviz BP, de Brito Simith DDJ, Fernandes MEB (2020) Natural recovery of the crab *Ucides cordatus* (Ocypodidae) in replanted mangroves on the Brazilian Amazon. Coastal Wetlands 40, 2367–2379.

[r57] Diele K, Koch V, Abrunhosa FA, de Farias Lima J, Simith, DJB (2010) The Brachyuran crab community of the Caeté Estuary, North Brazil: species richness, zonation and abundance. In Saint-Paul U and Schneider H (eds.), *Mangrove Dynamics and Management in North Brazil. Ecological Studies* , vol 211. Berlin, Heidelberg: Springer. 10.1007/978-3-642-13457-9_16

[r58] Diniz CL, Nerino G, Rodrigues J, Sadeck L, Adami M and Souza-Filho PWM (2019) Brazilian mangrove status: three decades of satellite data analysis. Remote Sensing 11, 808. 10.3390/rs11070808

[r59] Diniz UM, Nadia TL, Mello MAR and Machado IC (2022) Few plants and one dominant fly shape a unique pollination network in a neotropical mangrove. Aquatic Botany 180, 103526. 10.1016/j.aquabot.2022.103526

[r60] El-Tarabily KA, Sham A, Elbadawi AA, Hassan AH, Alhosani BKK, El-Esawi MA, AlKhajeh AS and AbuQamar SF (2021) A Consortium of rhizosphere-competent actinobacteria exhibiting multiple plant growth-promoting traits improves the growth of *Avicennia marina* in the United Arab Emirates. Frontiers in Marine Science 8, 715123. 10.3389/fmars.2021.715123

[r61] Eyre BD and Ferguson AJP (2005) Benthic metabolism and nitrogen cycling in a subtropical east Australian estuary (Brunswick): temporal variability and controlling factors. Limnology and Oceanography 50, 81–96.

[r62] Farooqui Z, Shafique S, Khan KL, Ali A, Iqbal P, Pirzada A and Siddiqui PJA (2012) Assessment of litter production in semi-arid mangrove forests near active Indus River mouth (Hajambro Creek) and Karachi backwaters, Pakistan. Pakistan Journal of Botany 44, 1763–1768.

[r63] Farrer EC, Van Bael SA, Clay K and Smith MKH (2022) Plant microbial symbioses in coastal systems: their ecological importance and role in coastal restoration. Estuaries and Coasts 45, 1805–1822. 10.1007/s12237-022-01052-2

[r64] Feller IC (2002) The role of herbivory by wood-boring insects in mangrove ecosystems in Belize. Oikos 97, 153–176. 10.1034/j.1600-0706.2002.970202.x

[r65] Fernandes MEB, Nascimento AAM and Carvalho ML (2007) Estimativa da produção anual de serapilheira dos bosques de mangue no Furo Grande, Bragança-Pará. Revista Árvore 31, 949–958. 10.1590/S0100-67622007000500019

[r66] Ferrante L and Fearnside PM (2019) Brazil’s new president and ‘ruralists’ threaten Amazonia’s environment, traditional peoples and the global climate. Environmental Conservation 46, 261–263. 10.1017/s0376892919000213

[r67] Ferreira AC, Alencar CERD and Bezerra LEA (2019b) Interrelationships among ecological factors of brachyuran crabs, trees and soil in mangrove community assemblage in Northeast Brazil. Community Ecology 20, 277–290. 10.1556/168.2019.20.3.8

[r68] Ferreira AC, Ashton EC, Ward RD, Hendy I and Lacerda LD (2024) Mangrove biodiversity and conservation: setting key functional groups and risks of climate-induced functional disruption. Diversity 16, 423. 10.3390/d16070423

[r69] Ferreira AC, Bezerra LEA and Mathews-Cascon H (2019a). Aboveground stock in a restored Neotropical mangrove: influence of management and brachyuran crab assemblage. Wetlands Ecology and Management 27, 223–242. 10.1007/s11273-019-09654-7

[r70] Ferreira AC, Freire FAM, Rodrigues JVM and Bezerra LEA (2022a) Mangrove recovery in semiarid coast shows increase in ecological processes from biotic and abiotic drivers in response to hydrological restoration. Wetlands 42, 1–19. 10.1007/s13157-022-01603-0

[r71] Ferreira AC, Ganade G and Attayde JL (2015) Restoration versus natural regeneration in a neotropical mangrove: effects on plant biomass and crab communities. Ocean and Coastal Management 110, 38–45. 10.1016/j.ocecoaman.2015.03.006

[r72] Ferreira AC and Lacerda LD (2016a) Degradation and conservation of Brazilian mangroves, status and perspective. Ocean and Coastal Management 125, 38–46. 10.1016/j.ocecoaman.2016.03.011

[r73] Ferreira AC and Lacerda LD (2016b) Reply to On the impact of the Brazilian Forrest Code on mangroves: a comment to Ferreira & Lacerda (2016) by Ronaldo Ruy Oliveira Filho et al. Ocean and Coastal Management 132, 170–171. 10.1016/j.ocecoaman.2016.08.003

[r74] Ferreira AC, Lacerda LD, Rodrigues JVM and Bezerra LEA (2023) New contributions to mangrove rehabilitation/restoration protocols and practices. Wetlands Ecology and Management 31, 89–114. 10.1007/s11273-022-09903-2

[r75] Ferreira AC and Sankarankutty C (2002) Estuarine Carcinofuna (Decapoda) of Rio Grande do norte, Brazil. Nauplius 2, 121–129.

[r76] Ferreira TO, Nóbrega GN, Queiroz HM, Souza Júnior VS, Barcellos D and Ferreira AD (2021) Windsock behavior: climatic control on iron biogeochemistry in tropical mangroves. Biogeochemistry 156, 437–452. 10.1007/s10533-021-00858-9

[r77] Ferreira TO, Otero XL, Vidal-Torrado P and Macías F (2007) Redox processes in mangrove soils under *Rhizophora mangle* in relation to different environmental conditions. Soil Science Society of America Journal 71, 484–491. 10.2136/sssaj2006.0078

[r78] Ferreira TO, Queiroz HM, Nóbrega GN, de Souza Júnior VS, Barcellos D, Ferreira AD and Otero XL (2022b) Litho-climatic characteristics and its control over mangrove soil geochemistry: a macro-scale approach. Science of the Total Environment 811, 152152. 10.1016/j.scitotenv.2021.152152 34919924

[r79] Filho CS, Tagliaro CH and Beasley CR Colin R. (2008) Seasonal abundance of the shipworm N*eoteredo reynei* (Bivalvia, Teredinidae) in mangrove driftwood from a northern Brazilian beach. Iheringia. Série Zoologia 98(1), 17–23.

[r80] Fusi M, Booth JM, Marasco R, Merlino G, Garcias-Bonet N, Barozzi A, Garuglieri E, Mbobo T, Diele K, Duarte CM and Daffonchio D (2022) Bioturbation Intensity modifies the sediment microbiome and biochemistry and supports plant growth in an arid mangrove system. Microbiology Spectrum 10, e0111722. 10.1128/spectrum.01117-22 35647697 PMC9241789

[r81] Garcia TM, Matthews-Cascon H, Schettini CAF, Matsumura-Tundisi JG and Neumann-Leitão S (2020) Mesozooplankton community of a dammed estuary in Brazilian semi-arid region. Cahiers de Biologie Marine 61, 149–158. 10.21411/CBM.A.3B7F837B

[r82] Gardunho DCL, Virgulino Júnior PCC and Fernandes MEB (2023) Avaliação das atividades de reflorestamento em áreas degradadas de manguezal. In Fernandes MEB (ed.), Projeto Mangues da Amazônia, uma abordagem socioambiental. Bragança: Laboratório de Ecologia de Manguezal, p. 148.

[r83] Gilman EL, Ellison J, Duke NC and Field C (2008) Threats to mangroves from climate change and adaptation options: a review. Aquatic Botany 89, 237–250. 10.1016/j.aquabot.2007.12.009

[r84] Godoy MDP and Lacerda LD (2014) River-island morphological response to basin Land-use changes within the Jaguaribe river estuary, NE Brazil. Journal of Coastal Research. 30, 399–410. 10.2112/JCOASTRES-D-13-00059.1

[r85] Godoy MDP and Lacerda LD (2015) Mangroves response to climate change: a review of recent findings on mangrove extension and distribution. Anais da Academia Brasileira de Ciências 87, 651–667. 10.1590/0001-3765201520150055 25993360

[r86] Godoy MDP, Meireles AJA and Lacerda LD (2018) Mangrove response to land use change in estuaries along the semiarid coast of Ceará, Brazil. Journal of Coastal Research 34, 524–533. 10.2112/JCOASTRES-D-16-00138.1

[r87] Gomes LEO, Vescovi LC and Bernadino AF (2021) The collapse of mangrove litterfall production following a climate-related forest loss in Brazil. Marine Pollution Bulletin 162, 111910. 10.1016/j.marpolbul.2020.111910 33338926

[r88] Gonçalves ASC, Fernandes MEB and Carvalho ML (2006) Variação anual da produção de serapilheira em bosques de mangue no Furo Grande, Bragança, Pará. Boletim do Museu Paraense Emílio Goeldi 2, 69–76.

[r89] Hatje V, Masqué P, Patire VF, Dórea A and Barros F (2021) Blue carbon stocks, accumulation rates, and associated spatial variability in Brazilian mangroves. Limnology and Oceanography 66, 321–334. 10.1002/lno.11607

[r90] Hendy I, Michie L and Taylor BW (2014) Habitat creation and biodiversity maintenance in mangrove forests: teredinid bivalves as ecosystem engineers. PeerJ 2, e591. 10.7717/peerj.591 25276505 PMC4178455

[r91] Hendy IW, Shipway JR, Tupper M, Etxabe AG, Ward RD and Cragg SM (2022) Biodegraders of large woody debris across a tidal gradient in an Indonesian mangrove ecosystem. Frontiers in Forests and Global Change 5, 852217. 10.3389/ffgc.2022.852217

[r92] Hogarth PJ (1999) The Biology of Mangroves. London: Oxford University Press.

[r93] Holguín G, Vazquez P and Bashan Y (2001) The role of sediment microorganisms in the productivity, conservation, and rehabilitation of mangrove ecosystems: an overview. Biology and Fertility of Soils 33, 265–278. 10.1007/s003740000319

[r94] ICMBio (2018) Atlas dos Manguezais do Brasil. Instituto Chico Mendes de Conservação da Biodiversidade. Available at https://www.gov.br/icmbio/pt-br/centrais-de-conteudo/publicacoes/atlas-1/atlas_dos_manguezais_do_brasil.pdf/view

[r95] ICMBio (2019) Portaria N° 647, de 30 de outubro de 2019 – Plano de Ação Nacional para a Conservação das Espécies Ameaçadas e de Importância Socioeconômica do Ecossistema Manguezal – PAN Manguezal. Available at https://www.gov.br/icmbio/pt-br/assuntos/biodiversidade/pan/pan-manguezal/1-ciclo/pan-manguezal-portaria-aprovacao-e-gat.pdf

[r96] Jennerjahn TC, Gilman E, Krauss KW, Lacerda LD, Nordhaus I and Wolanski E (2017) Climate Change. In Rivera-Monroy VH, Lee SY, Kristensen E and Twilley RR (eds.), Mangrove Ecosystems: A Global Biogeographic Perspective, Chapter 7, pp. 211–244. Berlin: Springer Verlag 10.1007/978-3-319-62206-4_7

[r97] Jimenez LCZ, Queiroz HM, Otero XL, Nóbrega GN and Ferreira TO (2021) Soil organic matter responses to mangrove restoration: a replanting experience in Northeast Brazil. International Journal of Environmental Research and Public Health 18, 8981. 10.3390/ijerph18178981 34501570 PMC8431229

[r98] Kathiresan K and Rajendran N (2005) Coastal mangrove forests mitigated tsunami. Estuarine, Coastal and Shelf Science 65, 601–606. 10.1016/j.ecss.2005.06.022

[r99] Kauffman JB, Bernardino AF, Ferreira TO, Bolton NW, Gomes LEO and Nobrega GN (2018b) Shrimp ponds lead to massive loss of soil carbon and greenhouse gas emissions in northeastern Brazilian mangroves. Ecology and Evolution 8, 5530–5540. 10.1002/ece3.4079 29938071 PMC6010805

[r100] Kauffman JB, Bernardino AF, Ferreira TO, Giovannoni LRO, Gomes LE, Romero DJ, Jimenez LCZ and Ruiz F (2018a) Carbon stocks of mangroves and salt marshes of the Amazon region, Brazil.Biology Letters 14: 20180208. 10.1098/rsbl.2018.0208 30185605 PMC6170755

[r101] Kjerfve B and Lacerda LD (1993) Mangroves of Brazil. In Mangrove Ecosystems Technical Reports, ITTO TS-13 2, Okinawa: International Society for Mangrove Ecosystems, pp. 245–272.

[r102] Komiyama A, Poungparn S, Umnouysin S, Rodtassana C, Pravinvongvuthi T, Noda T and Kato S (2019) Occurrence of seasonal water replacement in mangrove soil and the trunk growth response of *Avicennia alba* related to salinity changes in a tropical monsoon climate. Ecological Research 34, 428–439. 10.1111/1440-1703.12005

[r103] Krauss KW, Lovelock CE, McKee KL, López-Hofman L, Ewe SML and Sousa WP (2008) Environmental drivers in mangrove establishment and early development: a review. Aquatic Botany 89, 105–127. 10.1016/j.aquabot.2007.12.014

[r104] Kristensen E (2008) Mangrove crabs as ecosystem engineers; with emphasis on sediment processes. Journal of Sea Research 59, 30–43. 10.1016/j.seares.2007.05.004

[r105] Kumar A and Ramanathan A (2015) Speciation of selected trace metals (Fe, Mn, Cu and Zn) with depth in the sediments of Sundarban mangroves: India and Bangladesh. Journal of Soils and Sediments 15, 2476–2486. 10.1007/s11368-015-1257-5

[r106] Lacerda LD (2002) Mangrove Ecosystems: Function and Management. Berlin: Springer Verlag, p. 292.

[r107] Lacerda LD (2018) Burial of mangroves by mobile dunes: a climate change threat in semiarid coasts. ISME/GLOMIS Electronic Journal 16(2), 6–10.

[r108] Lacerda LD, Borges R and Ferreira AC (2019) Neotropical mangroves: conservation and sustainable use in a scenario of global climate change. Aquatic Conservation: Marine and Freshwater Ecosystems 29, 1347–1364. 10.1002/aqc.3119

[r109] Lacerda LD, Cavalcante IKB, Soares AA and Marins RV (2024). Mobility, bioavailability and distribution of Fe and Cu in mangroves (*Avicennia schaueriana* and *Rhizophora mangle*) from a semiarid coast in NE Brazil. Anais da Academia Brasileira de Ciências 96, e20231075. 10.1590/0001-3765202420231075 38747797

[r110] Lacerda LD, Dias FJS, Marins RV, Soares TM, Godoy JM and Godoy MLDP (2013) Pluriannual watershed discharges of Hg into tropical semi-arid estuary of the Jaguaribe River, NE Brazil. J. Braz. Chem. Soc. 24, 1719–1731. 10.5935/0103-5053.20130216

[r111] Lacerda LD, Ferreira AC, Borges R and Ward RD (2022a) Mangroves of Brazil. In Das SC, Ashton E and Thammineni P (eds.), Mangroves: Biodiversity, Livelihoods and Conservation. Berlin: Springer Verlag, pp. 521–563. 10.1007/978-981-19-0519-3_20

[r112] Lacerda LD, Marins RV and Dias FJS (2020) An arctic paradox: response of fluvial Hg inputs and its bioavailability to global climate change in an extreme coastal environment. Frontiers in Earth Science 8, 93. 10.3389/feart.2020.00093

[r113] Lacerda LD, Menezes MOT and Molisani MM (2007) Changes in mangrove extension at the Pacoti River estuary, CE, NE Brazil due to regional environmental changes between 1958 and 2004. Biota Neotropica 7, 1–6.

[r114] Lacerda LD and Miguens FC (2011) A ressurreição do metal: a contaminação em sedimentos de estuários e deltas. Ciência Hoje 47(287), 38–41.

[r115] Lacerda LD, Molisani MM, Sena D and Maia LP (2008) Estimating the importance of natural and anthropogenic sources on N and P emission to estuaries along the Ceará State Coast NE Brazil. Environmental Monitoring and Assessment 141, 149–164. 10.1007/s10661-007-9884-y 17876716

[r116] Lacerda LD, Rezende CE, José DMV, Francisco MCF, Wasserman JC and Martins JC (1986) Leaf chemical characteristics affecting herbivory in a New World mangrove forest. Biotropica 18, 350–355.

[r117] Lacerda LD, Ward R, Ferreira AC, Borges R, Godoy, MDP and Meireles J (2021) 20-years cumulative impact from shrimp farming on mangroves of Northeast Brazil. Frontiers in Forests and Global Change 4, 653096. 10.3389/ffgc.2021.653096

[r118] Lacerda LD, Ward RD, Borges R and Ferreira AC (2022b) Mangrove trace-metal biogeochemistry response to global climate change. Frontiers in Forests and Global Change 5, 817992. 10.3389/ffgc.2022.817992

[r119] Lee SY (2008) Mangrove macrobenthos: assemblages, services, and linkages. Journal of Sea Research 59, 16–29. 10.1071/MF97179

[r120] Lei P, Zhong H, Duan D and Pan K (2019) A review on mercury biogeochemistry in mangrove sediments: hotspots of methylmercury production? Science of the Total Environment 680, 140–150. 10.1016/j.scitotenv.2019.04.451 31112813

[r121] Lewis III RR (2005) Ecological engineering for successful management and restoration of mangrove forests. Ecological Engineering 24, 403–418. 10.1016/j.ecoleng.2004.10.003

[r122] Lewis III RR (2009) Methods and criteria for successful mangrove forest restoration. In Perillo GME, Wolanski E, Cahoon DR and Brinson MM (eds.), Coastal Wetlands, an Integrated Ecosystem Approach. London: Elsevier. pp. 787–800.

[r123] Lewis RR and Gilmore RG (2007) Important considerations to achieve successful mangrove forest restoration with optimum fish habitat. Bulletin of Marine Science 80, 823–837.

[r124] Lira MGS, Berbel-Filho WM, Espírito-Santo HMV, Tatarenkov A, Avise JC, Leaniz CG, Consuegra S and Lima SMQ (2021) Filling the gaps: phylogeography of the self-fertilizing *Kryptolebias* species (Cyprinodontiformes: Rivulidae) along South American mangroves. Journal of Fish Biology 99, 644–655. 10.1111/jfb.14753 33846974

[r125] Losekann C and Paiva RL (2024) Brazilian environmental policy: shared responsibility and dismantling. Ambiente & Sociedade 27, 1–21. 10.1590/1809-4422asoc0176r4vu27l1oa

[r126] Lovelock CE, Krauss KW, Osland MJ, Reef R and Ball MC (2016) The physiology of mangrove trees with changing climate. In Goldstein G and Santiago L (eds.), Tropical Tree Physiology: Adaptations and Responses in a Changing E. New York, NY: Springer, pp 149–179.

[r127] Lu W, Xiao J, Cui X, Xu F, Lin G and Lin G (2019) Insect outbreaks have transient effects on carbon fluxes and vegetative growth but longer-term impacts on reproductive growth in a mangrove forest. Agricultural and Forest Meteorology 279, 107747. 10.1016/j.agrformet.2019.107747

[r128] Machado W, Gueiros BB, Lisboa Filho SD and Lacerda LD (2005) Trace metals in mangrove seedlings: The role of iron plaque formation. Wetlands Ecology and Management 13, 199–206. 10.1007/s11273-004-9568-0

[r129] Machado W, Moscatelli M, Rezende LG and Lacerda LD (2002) Mercury, zinc and copper accumulation in mangrove sediments affected by landfill wastewater. Environmental Pollution 120, 455–461. 10.1016/S0269-7491(02)00108-2 12395859

[r130] Madi APLM, Boeger MRT and Reissmann CB (2015) Distribution of Cu, Fe, Mn, and Zn in two mangroves of Southern Brazil. Brazilian Archives of Biology and Technology 58, 970–976. 10.1590/S1516-89132015060255.

[r131] Maia LP, Freire GSS and Lacerda LD (2005) Accelerated dune migration and sand transport during El Niño events along the NE Brazilian coast. Journal of Coastal Research 21, 1121–1126. 10.2112/03-702A.1.

[r132] Maia LP, Lacerda LD, Monteiro LHU and Souza GM (2006) *Atlas dos Manguezais do Nordeste do Brasil: Avaliação das Áreas de Manguezais dos Estados do Piauí, Ceará, Rio Grande do Norte, Paraíba e Pernambuco.* Secretaria do meio Ambiente do Estado do Ceará, Fortaleza, p. 150.

[r133] Maia RC, Rosa Filho JS, de Almeida Rocha-Barreira C, Matthews-Cascon H, dos Santos ES, David HN and Matos AS (2018) Benthic estuarine assemblages of the Northeastern Brazil Marine Ecoregion. In Lana PC and Bernardino AF(eds.), Brazilian Estuaries: A Benthic Perspective. Berlin: Springer Verlag, pp. 75–94. 10.1007/978-3-319-77779-53

[r134] Maia RC, Sousa KNS, Benevides JAJ, Amorim VG and Sousa RM (2019) Impactos ambientais em manguezais no Ceará: Causas e consequências. *Conexões* Ciênccia e Tecnologia 13, 69–77. 10.21439/conexoes.v13i5.1797

[r135] Makowski C and Finkl CW (2018) Threats to Mangrove Forests: Hazards, Vulnerability, and Management. Coastal Research Library. Berlin: Springer, p. 724. 10.1007/978-3-319-73016-5

[r136] Maldonado-López Y, Vaca-Sánchez MS, Canché-Delgado A, García-Jaín SE, González-Rodríguez A, Cornelissen T and Cuevas-Reyes P (2019) Leaf herbivory and fluctuating asymmetry as indicators of mangrove stress. Wetlands Ecology and Management 27, 571–580. 10.1007/s11273-019-09678-z

[r137] Maltchik L and Medeiros ESF (2006) Conservation importance of semi-arid streams in north-eastern Brazil: implications of hydrological disturbance and species diversity. Aquatic Conservation: Marine and Freshwater Ecosystems 16, 665–677. 10.1002/aqc.805

[r138] Marengo JA, Alves LM, Alvalá RCS, Cunha AP, Brito S and Moraes OLL (2018) Climatic characteristics of the 2010-2016 drought in the semiarid Northeast Brazil region. Anais da Academia Brasileira de Ciências 90, 1973–1985. 10.1590/0001-3765201720170206 28813107

[r139] Marengo JA, Cunha AP, Nobre CA, Ribeiro Neto GG, Magalhaes AR, Torres RR, Sampaio G, Alexandre F, Alves LM, Cuartas LA, Deusdará KRL and Álvala RCS (2020) Assessing drought in the drylands of northeast Brazil under regional warming exceeding 4°C. Natural Hazards 103, 2589–2611, 10.1007/s11069-020-04097-3.

[r221] Marins RV, Lacerda LD, Goncalves GO and Paiva EC (1997) Effects of root metabolism on the postdepositional mobilization of mercury in salt marsh soils. Bulletin of Environmental Contamination & Toxicology 58: 733–738. 9115135 10.1007/s001289900394

[r140] Marins RV, Freire GSS, Maia LP and Lacerda LD (2002) Regional assessment tables RA 3: north-eastern Brazil tectonically passive coast. In Lacerda LD, Kremer HH, Kjerfve B, Salomons W, Marshall-Crossland JI and Crossland JC (eds.), South American Basins: LOICZ Global Change Assessment and Synthesis of River Catchment – Coastal Sea Interaction and Human Dimensions. Texel: LOICZ Reports & Studies, No. 21, pp. 169–172.

[r141] Marins RV, Lacerda LD, Abreu IM and Dias FJS (2003). Efeitos da açudagem no Rio Jaguaribe. Ciência Hoje 33(197), 66–70.

[r142] Marins RV, Lacerda LD, Araújo ICS, Fonseca LV and Silva ATF (2020) Phosphorus and suspended matter retention in mangroves affected by shrimp farm effluents in NE Brazil. Anais da Academia Brasileira de Ciências 92(3), e20200758 10.1590/0001-3765202020200758 33111824

[r143] Marins RV, Paula Filho J, Eschrique SA and Lacerda LD (2011) Anthropogenic sources and distribution of phosphorus in sediments from the Jaguaribe River estuary, NE, Brazil, Brazilian Journal of Biology 71, 673–678. 10.1590/S1519-69842011000400011 21881790

[r144] Marochi MZ, Grande FR, Pardo JCF, Montenegro A and Costa TM (2022) Marine heatwave impacts on newly hatched planktonic larvae of an estuarine crab. *Estuarine, Coastal and Shelf* Science **278**(2), 108122. 10.1016/j.ecss.2022.108122

[r145] Mattos PP, Nobre IM and Aloufa MAI (2011) Reserva de Desenvolvimento Sustentável: avanço na concepção de áreas protegidas? *Sociedade & N*atureza 3, 409–422. 10.1590/S1982-45132011000300004

[r146] McKee KL (1995) Mangrove species distribution and propagule predation in Belize: an exception to the dominance-predation hypothesis. Biotropica 27, 334–345. 10.2307/2388919

[r147] McLeod E and Salm RV (2006) *Managing Mangroves for Resilience to Climate Change.* World Conservation Union (IUCN), Gland. Available at https://portals.iucn.org/library/efiles/documents/2006-041.pdf

[r148] MDSCF (2016) Ministério do Desenvolvimento Social e Combate à Fome. *Estudo Técnico n. 08/2016. Programa Bolsa Verde: estratégia avaliativa e primeiros resultados acerca do desenho, perfil dos beneficiários e percepções de gestores do programa.* Available at https://aplicacoes.mds.gov.br/sagi/pesquisas/documentos/estudo/127.pdf

[r149] Mehlig U (2001) Aspects of Tree Primary Production in an Equatorial Mangrove Forest in Brazil, PhD Thesis. Bremen, Germany: University of Bremen, p. 151.

[r150] Menezes MPM and Mehlig U (2005) Massive defoliation of *Avicennia germinans* (L.) Stearn 1958 (Avicenniaceae) by *Hyblaea puera* (Lepidoptera: Hyblaeidae), in mangroves of Bragança, Pará State, Brazil. Boletim do Museu Paraense Emílio Goeldi. Ciências Naturais 1, 221–226.

[r151] Meng X, Xia P, Li Z and Meng D (2017) Mangrove development and its response to Asian monsoon in the Yingluo Bay (SW China) over the last 2000 years. Estuaries and Coasts 40, 540–552. 10.1007/s12237-016-0156-3

[r152] MMA (2015) Ministério do Meio Ambiente - *Plano de ação nacional para a conservação das espécies ameaçadas e de importância socioeconômica do ecossistema manguezal.* Available at https://www.gov.br/icmbio/pt-br/assuntos/biodiversidade/pan/pan-manguezal

[r153] Molisani MM, Cruz ALV and Maia LP (2006) Estimation of the freshwater river discharge to estuaries in. Arquivos de Ciências do Mar 39, 53–60. 10.32360/acmar.v39i1-2.6173

[r154] Moncunill DF (2006) The rainfall trend over Ceará and its implications. In Proceedings of 8th ICSHMO. São José dos Campos: Instituto Nacional de Pesquisas Espaciais, pp. 315–323.

[r155] Moomaw WR, Chmura GL, Davies GT, Finlayson CM, Middleton BA, Natali SM, Perry JE, Roulet N and Sutton-Grier AE (2018) Wetlands in a changing climate: science, policy and management. Wetlands 38, 183–205. 10.1007/s13157-018-1023-8

[r156] Morgado F, Santos RMAL, Sampaio D, LACERDA LD, Soares AMVM, Vieira, HC and Abreu S (2021) Chronological trends and mercury bioaccumulation in na aquatic semiarid ecosystem under a global climate change scenario in the Northeastern coast of Brazil. Animals 11, 2402. 10.3390/ani11082402 34438859 PMC8388643

[r157] Mounier SJ, Lacerda LD and Marins RV (2018) Determining the influence of urbanization on mangrove zones of Northeastern Brazil: characterization of Ceará State coastal zone organic matter inputs. In Makowski C (ed.), Threats to Mangrove Forests. Cham: Springer, pp. 199–222.

[r158] Mourão JS, Baracho RL, Martel G, Barboza RRD and Faria Lopes S (2020) Local ecological knowledge of shellfish collectors in an extractivist reserve, Northeast Brazil: implications for co-management. Hydrobiologia 847, 1977–1997. 10.1007/s10750-020-04226-w

[r159] Murugesan P, Sarathy PP, Muthuvelu S and Mahadevan G (2018) Diversity and distribution of polychaetes in mangroves of East Coast of India. In Sharma SD (ed.), Mangrove Ecosystem Ecology and Function, London: IntechOpen. pp. 107–130. 10.5772/intechopen.78332

[r160] Nadia TL and Machado IC (2014) Wind pollination and propagule formation in *Rhizophora mangle* L. (Rhizophoraceae): resource or pollination limitation?. Anais da Academia Brasileira de Ciências 86, 101712e. 10.1590/0001-37652014101712 24804313

[r161] Nagelkerken I, Blaber SJM, Bouillon S, Green P, Haywood M, Kirton LG, Meynecke JO, Pawlik J, Penrose HM, Sasekumar A and Somerfield PJ (2008) The habitat function of mangroves for terrestrial and marine fauna: a review. Aquatic Botany 89, 155–185. 10.1016/j.aquabot.2007.12.007

[r162] Nascimento RESA, Mehlig U and Menezes MPM (2006) Produção de serapilheira em um fragmento de bosque de terra firme e um manguezal vizinhos na península de Ajuruteua, Bragança, Pará. Boletim do Museu Paraense Emílio Goeldi 1, 71–76.

[r163] Nguyen H-H, Nghia NH, Nguyen HTT, Le AT, Tran, LTN, Duong LVK, Bohm S, and Furniss MJ (2020) Classification methods for mapping mangrove extents and drivers of change in Thanh Hoa Province, Vietnam during 2005-2018. Forestry and Society 4(1), 225–242. 10.24259/fs.v4i1.9295

[r164] Nóbrega GN, Ferreira TO, Romero RE, Marques AGB and Otero XL (2013) Iron and sulfur geochemistry in semi-arid mangrove soils (Ceará, Brazil) in relation to seasonal changes and shrimp farming effluents. Environmental Monitoring and Assessment 185, 7393–7407 10.1007/s10661-013-3108-4 23355026

[r165] Nóbrega GN, Ferreira TO, Siqueira Neto M, Mendonça ES, Romero RE and Otero XL (2019) The importance of blue carbon soil stocks in tropical semiarid mangroves: a case study in Northeastern Brazil. Environment and Earth Science 78, 1–10. 10.1007/s12665-019-8368-z

[r166] Nozarpour R, Shojaei MG, Naderloo R and Nasi F (2023) Crustaceans functional diversity in mangroves and adjacent mudflats of the Persian Gulf and Gulf of Oman. Marine Environmental Research 186, 105919. 10.1016/j.marenvres.2023.105919 36801504

[r167] Nurdiani R and Zeng C (2007) Effects of temperature and salinity on the survival and development of mud crab, *Scylla serrata* (Forsskål), larvae. Aquaculture Research 8, 1529–1538. 10.1111/j.1365-2109.2007.01810.x

[r168] Orélis-Ribeiro R, Boeger WA, Vicente VA, Chammas M and Ostrensky A (2011) Fulfilling Koch’s postulates confirms the mycotic origin of Lethargic Crab Disease. Antonie Van Leeuwenhoek 99(3), 601–608. 10.1007/s10482-010-9531-4 21152982

[r169] Ottonelli J and Mariano JL (2014) Pobreza multidimensional nos municípios da Região Nordeste. Revista de Administração Pública 48, 1253–1279. 10.1590/0034-76121724

[r170] Passos T, Penny D, Sanders C, França E, Oliveira T, Santos L and Barcellos R (2021) Mangrove carbon and nutrient accumulation shifts driven by rapid development in a tropical estuarine system, northeast Brazil. Marine Pollution Bulletin 166, 112219. 10.1016/j.marpolbul.2021.112219 33690084

[r171] Pereira LS, Chaves FO and Soares MLG (2023) Herbivory in southeastern Brazilian mangroves: An analysis of 11 years of litterfall monitoring. Aquatic Botany 186, 103634.

[r172] Perry DM and Brusca RC (1989) Effect of the root-boring isopod *Sphaeroma peruvianum* on red mangrove forests. Marine Ecology Progress Series 57, 287–292 10.3354/meps057287

[r173] Portela MGT, Espinola GM, Vallares GS, Amorin JVA and Frota JCO (2020) Vegetation biomass and carbon stocks in the Parnaíba River Delta, NE Brazil. Wetlands Ecology and Management 28, 607–622. 10.1007/s11273-020-09735-y

[r174] Pörtner HO, Scholes, RJ, Arneth A, Barnes DKA, Burrows MT, Diamond SE, Duarte CM, Kiessling W, Leadley P, Managi S, McElwee P, Midgley G, Ngo HT, Obura D, Pascual U, Sankaran M, Shin Y-J and Val V (2023) Overcoming the coupled climate and biodiversity crises and their societal impacts. Science 380, 6642. 10.1126/science.abl488 37079687

[r175] Proisy C, Gratiot N, Anthony EJ, Gardel A, Fromard F and Heuret P (2009) Mud bank colonization by opportunistic mangroves: A case study from French Guiana using lidar data. Continental Shelf Research 29, 632–641. 10.1016/j.csr.2008.09.017

[r176] Queiroz HM, Artur AG, Taniguchi CAK, Silveira MRS da, Nascimento JC do, Nóbrega G.N., Otero XL and Ferreira TO (2019) Hidden contribution of shrimp farming effluents to greenhouse gas emissions from mangrove soils. Estuarine, Coastal and Shelf Science 221, 8–14. 10.1016/j.ecss.2019.03.011

[r177] Queiroz HM, Ferreira TO, Taniguchi CAK, Barcellos D., Nascimento JC do, Nóbrega GN, Otero XL and Artur AG. (2020) Nitrogen mineralization and eutrophication risks in mangroves receiving shrimp farming effluents. Environmental Science and Pollution Research 27, 34941–34950. 10.1007/s11356-020-09720-1 32583107

[r178] Queiroz LS, Rossi S, Calvet-Mir, L, Ruiz-Mallén I, García-Betorz S, Salvà-Prat J and Meireles AJAM (2017) Neglected ecosystem services: Highlighting the socio-cultural perception of mangroves in decision-making processes. Ecosystem Services 26, 137–145 10.1016/j.ecoser.2017.06.013

[r179] Rabelo-Mochel F (1997) Mangroves on São Luís Island, Maranhão, Brazil. In Lacerda LD, Kjerfve B and Diop S (eds.), Mangrove Ecosystem Studies in Latin America and Africa. Paris: UNESCO. pp. 145–154.

[r180] Rosa Filho JS, Pereira LCC, Aviz D, Braga CF, Monteiro MC, Costa RAM, Asp NE and Beasley CR (2018) Benthic estuarine assemblages of the Brazilian North Coast (Amazonia Ecoregion). In Lana P and Bernardino A (eds.), Brazilian Estuaries. Brazilian Marine Biodiversity. Cham: Springer. 10.1007/978-3-319-77779-5_2

[r181] Ross MS, O’Brien Jj, Ford RG, Zhang K and Morkill A (2009) Disturbance and the rising tide: the challenge of biodiversity management on low-island ecosystems. Frontiers in Ecology and the Environment 7, 471–478. 10.1890/070221

[r182] Rovai AS, Twilley RR, Worthington TA and Riul P (2022) Brazilian mangroves: blue carbon hotspots of national and global relevance to natural climate solutions. Frontiers in Forests and Global Change 4,787533. 10.3389/ffgc.2021.78753

[r183] Saderne V, Cusack, M, Almahasheer H., Serrano O, Masqué P, Arias-Ortiz A, Krishnakumar PK and Rabaoui L (2018) Accumulation of carbonates contributes to coastal vegetated ecosystems keeping pace with sea level rise in an arid region (Arabian Peninsula). Journal of Geophysical Research – Biogeosciences 123, 1498–1510. 10.1029/2017JG004288

[r184] Sanchez-Carrillo S, Sanchez-Andre R, Alatorre LC, Angeler DG, Lvarez-Cobelas MA and Arreola-Lizarraga JA (2009) Nutrient fluxes in a semi-arid microtidal mangrove wetland in the Gulf of California. Estuarine, Coastal and Shelf Science 82, 654–662. 10.1016/j.ecss.2009.03.002

[r185] Sanders CJ, Eyre BD, Santos IR, Machado W, Luiz-Silva W, Smoak JM, Breithaupt JL, Ketterer ME, Sanders L, Marotta H and Silva-Filho E (2014) Elevated rates of organic carbon, nitrogen, and phosphorus accumulation in a highly impacted mangrove wetland. Geophysical Research Letters 41, 2475–2480. 10.1002/2014GL059789

[r186] Santos IR, Beltrão NES and Trindade AR (2019) Carbono “azul” nos manguezais amazônicos: conservação e valoração econômica. Revista iberoamericana de Economía Ecológica 31, 18–28.

[r187] Schaefer CRGR, Lima H, Teixeira GW, Vale JF, Souza KW, Corrêa GR, Mendonça BAF, Amaral E, Campos MCC and Ruivo MLP (2017) Solos da região Amazônica. In Curi N, Ker JC, Novais RF, Vidal-Torrado P and Schaefer CRGR (eds.), Pedologia: Solos dos Biomas Brasileiros, 1st Ed. Sociedade Brasileira de Ciência do Solo, pp. 111–174.

[r188] Schumacher BA (2002) Methods for the Determination of Total Organic Carbon (TOC) in Soils and Sediments. Washington, DC: US Environmental Protection Agency, Office of Research and Development, Ecological Risk Assessment Support Center, p. 23.

[r189] Servino RN, Gomes LEO and Bernardino AF (2018) Extreme weather impacts on tropical mangrove forests in the Eastern Brazil Marine Ecoregion. Science of the Total Environment 628/629, 233–240. 10.1016/j.scitotenv.2018.02.068 29444481

[r190] Sheaves M (2009) Consequences of ecological connectivity: the coastal ecosystem mosaic. Marine Ecology Progress Series 391, 107–115. 10.3354/meps08121

[r191] Silva CAR, Lacerda LD, Ovalle ARC and Rezende CE (1998) The dynamics of heavy metals through litterfall in a red mangrove forest. Mangroves and Salt Marshes 2, 149–157.

[r192] Silva EV, Rabelo FDB and Cestaro LA (2020) Biogeography and ecology of the mangrove ecosystems from the semi-arid coast of the Northeast Brazil. RA’EGA 8, 22–41. 10.5380/raega.v49i0.65811

[r193] Silva Filho LA and Queiroz S (2011) Recuperação econômica e emprego formal: avaliação para o Nordeste brasileiro entre 2000 e 2008. Perspective Economics 7, 42–54. 10.4013/pe.2011.71.04

[r194] Silva Júnior JJ, Nicacio G and Rodrigues GG (2020) A carcinicultura nos manguezais do Nordeste brasileiro: problemáticas socioambientais nas comunidades tradicionais. Revista Movimentos Sociais e Dinâmicas Espaciais 9, 70. 10.46802/rmsde.v9i2.245816

[r195] Silva MH, Silva-Cunha MDGG, Passavante JZO, Grego CKS and Muniz K (2009) Estrutura sazonal e espacial do microfitoplâncton no estuário tropical do rio Formoso, PE, Brasil. Acta Botânica Brasílica 23, 355–368.

[r196] Silva RJR and Maia RC (2022) Leaf herbivory in a mangrove forest in Ceará, Brazil. Ciencia Florestal 32, 122–140. 10.5902/1980509843456

[r197] Singh M, Schwendenmann L, Wang G, Adame MF and Mandlate LJC (2022) Changes in mangrove carbon stocks and exposure to sea level rise (SLR) under future climate scenarios. Sustainability 14(7), 3873. 10.3390/su14073873

[r198] Sippo JZ, Maher DT, Tait DR, Holloway C and Santos IR (2016) Are mangroves drivers or buffers of coastal acidification? Global Biogeochemical Cycles 30, 753–766. 10.1002/2015GB005324

[r199] Smith III TJ, Chan HT, McIvor CC and Robblee MB (1989) Comparisons of seed predation in tropical tidal forests from three continents. Ecology 70, 146–151. 10.2307/1938421

[r200] Soares MO, Bezerra LEA, Copertino M, Lopes BD, Barros KVS, Rocha-Barreira CA, Maia RC, Beloto N and Cotovicz LC (2022). Blue carbon ecosystems in Brazil: Overview and an urgent call for conservation and restoration. Frontiers in Marine Science 9, 797411. 10.3389/fmars.2022.797411

[r201] Soares MO, Campos CC, Carneiro PBM, Barroso HS, Marins RV, Teixeira CEP, Menezes MOB, Pinheiro LS, Viana MB, Feitosa CV, Sánchez-Botero JI, Bezerra LEA, Rocha-Barreira CA, Matthews-Cascon H, Matos FO, Gorayeb A, Cavalcante MS, Moro MF, Rossi S, Belmonte G, Melo VMM, Rosado AS, Ramiresi G, Tavares TCL and Garcia TM (2021) Challenges and perspectives for the Brazilian semi-arid coast under global environmental changes. Perspectives in Ecology and Conservation 19, 267–278. 10.1016/j.pecon.2021.06.001

[r202] Soares-Filho B, Rajão R, Macedo M, Carneiro A, Costa W, Coe M, Rodrigues H and Alencar A (2014) Cracking Brazil’s Forest Code. Science 344, 363–364. 10.1126/science.1246663 24763575

[r203] Souza Filho PWM and Paradella WR (2003) Use of synthetic aperture radar for recognition of coastal geomorphological features, land use assessment and shoreline changes in Braganca Coast Param, North Brazil. Brazilian Academy of Sciences 75, 341–356. 10.1590/S0001-37652003000300007

[r204] Souza YG, Souza ACD, Saldanha DS and Costa DFS (2023) Serviço de regulação e manutenção do carbono na biomassa acima do solo em um manguezal semiárido Brasileiro. Geo UERJ 42, 74562. 10.12957/geouerj.2023.74562

[r205] Spedicato A, Zeppilli D, Thouzeau G and Michaud E (2023) Nematode diversity patterns in mangroves: a review of environmental drivers at different spatial scales. Biodiversity and Conservation 32, 1451–1471. 10.1007/s10531-023-02562-6

[r206] Suárez-Abelenda M, Ferreira TO, Camps-Arbestain M, Rivera-Monroy VH, Macías F, Nóbrega GN and Otero XL (2014) The effect of nutrient-rich effluents from shrimp farming on mangrove soil carbon storage and geochemistry under semi-arid climate conditions in northern Brazil. Geoderma 213, 551–559. 10.1016/j.geoderma.2013.08.007

[r207] Svavarsson J, Melckzedeck KW and Osore EO (2002) Does the wood-borer *Sphaeroma terebrans* (Crustacea) shape the distribution of the mangrove *Rhizophora mucronata*? Ambio 31, 574–579. 10.1579/0044-7447-31.7.574 12572825

[r208] Tavares FBR, Gil LM PR and Fontenele RES (2023) Carbono azul e recuperação de mangues: Potencialidades no ecossistema do Ceará. *Proc. 25th Encontr. Inter. Gestão Empres. Meio Ambien.* FEA/USPO (ENGEMA), pp. 1–16. Available at https://engemausp.submissao.com.br/25/anais/arquivos/554.pdf?v=1717079425

[r209] Tavares TCL, Bezerra WM, Normando LRO, Rosado AS and Melo VMM (2021) Brazilian semi-arid mangroves-associated microbiome as pools of richness and complexity in a changing world. Frontiers in Microbiology 12, 715991. 10.3389/fmicb.2021.715991 34512595 PMC8427804

[r210] Teles JN, Peres PA, Jimenez LCZ, Mantelatto FL and Quimbayo JP (2024) Congruence among taxonomic, functional, and phylogenetic diversity of mangrove crabs in the Southwestern Atlantic. Marine Biology 171, 25. 10.1007/s00227-023-04326-w

[r211] Ternes MLF, Freret-Meurer NV, Nascimento RL, Vidal MD and Giarrizzo T (2023) Local ecological knowledge provides important conservation guidelines for a threatened seahorse species in mangrove ecosystems. Frontiers in Marine Science 10, 1139368. 10.3389/fmars.2023.1139368

[r212] Valenti WC, Barros HP, Moraes-Valenti P, Bueno GW and Cavalli RO (2021) Aquaculture in Brazil: past, present and future. Aquaculture Reports 19, 100611. 10.1016/j.aqrep.2021.100611

[r213] Villamayor BMR, Rollon RN, Samson MS, Albano GMG and Primavera JH (2016) Impact of Haiyan on Philippine mangroves: implications to the fate of the widespread monospecifc *Rhizophora* plantations against strong typhoons. Ocean and Coastal Management 132, 1–14. 10.1016/j.ocecoaman.2016.07.011

[r214] Visschers LLB, Santos CD and Franco AMA (2022) Accelerated migration of mangroves indicate large-scale saltwater intrusion in Amazon coastal wetlands. Science of the Total Environment 836, 155679. 10.1016/j.scitotenv.2022.155679 35523322

[r215] Ward R, Friess D, Day R and Mackenzie R (2016) Impacts of climate change on global mangrove ecosystems: a regional comparison. Ecosystem Health and Sustainability 2, 1–25. 10.1002/ehs2.1211

[r216] Ward RD and Lacerda LD (2021) Response of mangrove ecosystems to sea level change. In Sidik F and Friess DA (eds.), Dynamic Sedimentary Environments of Mangrove Coasts. Amsterdam: Elsevier, pp. 235–253.

[r217] Ward RD, Lacerda LD, Cerqueira AC, Hugo V and Hernandez OC (2023) Impacts of sea level rise on mangroves in northeast Brazil. Estuarine, Coastal and Shelf Science 289, 108382. 10.1016/j.ecss.2023.108382

[r218] Warren JH and Underwood AJ (1986) Effects of burrowing crabs on the topography of mangrove swamps in New South Wales. Journal of Experimental Marine Biology and Ecology 102, 223–235. 10.1016/0022-0981(86)90178-4

[r219] Zamboni NS, Prudêncio MC, Amaro VE, Matos MFA, Verutes GM and Carvalho AR (2022) The protective role of mangroves in safeguarding coastal populations through hazard risk reduction: a case study in northeast Brazil. Ocean and Coastal Management 229, 106353. 10.1016/j.ocecoaman.2022.106353

