# Supplementary material for: Challenges to mangroves of the Semiarid Equatorial Coast of Brazil in the Anthropocene
Source: Camb Prism Coast Futur. 2024 Dec 4;2:e17. doi: 10.1017/cft.2024.16 (PMC12337612; doi:10.1017/cft.2024.16)
Supplement: Peer review history

# Editor report: Recommendation: Challenges to mangroves of the Semiarid Equatorial Coast of Brazil in the Anthropocene — R0/PR2

**DOI:** 10.1017/cft.2024.16.pr2

**Affiliations:** Institute for Coastal and Marine Research, Nelson Mandela University, South Africa

Dear authors

One reviewer has suggested that this is more of a review article or to trim down the article to only include the Biogeochemistry section. The other reviewer says that some sections are excessively long and need to be focused and made more concise.

Please work on shortening the manuscript.

One reviewer has uploaded the manuscript with annotated comments; please ensure that these are addressed.

Thanks 

# Editor report: Decision: Challenges to mangroves of the Semiarid Equatorial Coast of Brazil in the Anthropocene — R0/PR3

**DOI:** 10.1017/cft.2024.16.pr3

**Affiliations:** Geography, Cambridge University, United Kingdom of Great Britain and Northern Ireland

No accompanying comment.

# Editor report: Recommendation: Challenges to mangroves of the Semiarid Equatorial Coast of Brazil in the Anthropocene — R1/PR5

**DOI:** 10.1017/cft.2024.16.pr5

**Affiliations:** Institute for Coastal and Marine Research, Nelson Mandela University, South Africa

Thank-you authors for the revision; your article will make an excellent contribution to the global study of mangroves highlighting the special characteristics of the semiarid equatorial coast of Brazil. Reviewers inputs were adequately addressed with inclusion of suggested references and other edits relating to acidification, iron plaques, metal fixation and nutrient inputs.

There are a few edits in the word document for your attention; thereafter the manuscript will be ready for publication. Please correct the spelling of canopy in the left photo of Figure 6.

# Editor report: Decision: Challenges to mangroves of the Semiarid Equatorial Coast of Brazil in the Anthropocene — R1/PR6

**DOI:** 10.1017/cft.2024.16.pr6

**Affiliations:** Geography, Cambridge University, United Kingdom of Great Britain and Northern Ireland

No accompanying comment.

# Editor report: Recommendation: Challenges to mangroves of the Semiarid Equatorial Coast of Brazil in the Anthropocene — R2/PR8

**DOI:** 10.1017/cft.2024.16.pr8

**Affiliations:** Institute for Coastal and Marine Research, Nelson Mandela University, South Africa

Thank-you authors for the final small revisions; all have been attended to and the article is now ready for publication - well done.

# Editor report: Decision: Challenges to mangroves of the Semiarid Equatorial Coast of Brazil in the Anthropocene — R2/PR9

**DOI:** 10.1017/cft.2024.16.pr9

**Affiliations:** Geography, Cambridge University, United Kingdom of Great Britain and Northern Ireland

No accompanying comment.
